# CIP2A recruits SLX4-MUS81-XPF in mitosis and protects against replication stress

**DOI:** 10.1038/s44319-026-00807-3

**Published:** 2026-05-26

**Authors:** Alice Meroni, Annica Pellizzari, Nathalie Varisco, Francesco Leone, Giada Greco, Andrea Hänel, Pascale Brasier-Lutz, Isabell Witzel, Alessandro A Sartori, Manuel Stucki

**Affiliations:** 1 Department of Gynecology, University of Zurich and University Hospital of Zurich, Schlieren, Switzerland; 2 Institute of Molecular Cancer Research, University of Zurich, Zurich, Switzerland

**Keywords:** Cell Cycle, DNA Replication, Recombination & Repair

## Abstract

DNA replication stress can generate mitotic defects because incompletely replicated chromosomes or unresolved replication intermediates tether sister chromatids and hinder their segregation. We and others recently uncovered a mitotic role for the oncoprotein CIP2A, which promotes chromosome stability and is essential in homologous recombination–deficient (HRD) cells. However, how CIP2A safeguards mitotic genome integrity remains unclear. Here, we investigate the role of CIP2A in mitotic responses to replication stress. We show that replication stress induces a strong increase in CIP2A foci during mitosis, highlighting its involvement in processing under-replicated DNA. In wild-type cells, CIP2A is required for efficient recruitment of the scaffold SLX4 and the nucleases MUS81 and XPF to sites of under-replicated DNA. CIP2A loss disrupts this recruitment and leads to increased anaphase lagging chromosomes and micronuclei formation. CIP2A also contributes to mitotic DNA synthesis (MiDAS), although this varies across cell lines, indicating that MiDAS and SMX complex recruitment are not strictly coupled. Together, our findings identify CIP2A as a regulator of mitotic processing of under-replicated DNA and provide a framework for understanding context-dependent vulnerabilities in cancer cells.

## Synopsis

CIP2A safeguards mitotic genome integrity under replication stress by promoting recruitment of the scaffold SLX4 and the nucleases MUS81 and XPF to sites of under-replicated DNA in mitosis.

- CIP2A accumulates at sites of under-replicated DNA in mitosis and drives recruitment of the SLX4–MUS81–XPF complex.
- CIP2A’s contribution to mitotic DNA synthesis (MiDAS) varies across cell lines.
- Loss of CIP2A increases chromosomal instability under replication stress.

## Abstract (web summary)

CIP2A safeguards mitotic genome integrity under replication stress by promoting recruitment of the scaffold SLX4 and the nucleases MUS81 and XPF to sites of under-replicated DNA in mitosis.

## Graphical abstract

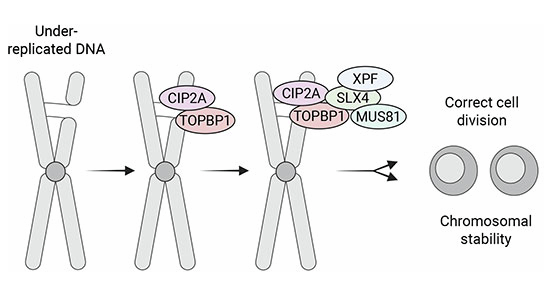

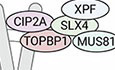

## Introduction

Faithful DNA replication is essential for genome stability, yet replication stress arising from DNA damage, replication fork stalling, or oncogene activation poses a significant threat to genomic integrity (Zeman and Cimprich, 2014). When replication is challenged during the S-phase, cells may enter mitosis with under-replicated DNA and single-strand DNA gaps. In this state, sister chromatids can remain physically linked, thereby hindering their proper segregation during anaphase. To prevent catastrophic chromosomal segregation errors, cells activate mitotic DNA synthesis (MiDAS), an atypical replication mechanism that resolves under-replicated DNA regions during early mitosis and promotes proper chromosome segregation (Bhowmick et al, 2023).

MiDAS is primarily observed under conditions of replication stress, particularly at late-replicating genomic regions such as common fragile sites (CFSs) (Le Beau et al, 1998). MiDAS is commonly induced by low doses of the DNA polymerase inhibitor aphidicolin, which slows replication and triggers transcription-replication conflicts at CFSs (Glover et al, 1984). Mechanistically, MiDAS involves coordinated actions of nucleases and polymerases to complete DNA synthesis, resembling break-induced replication (BIR) (Bhowmick et al, 2023). Under-replicated DNA sites are first marked by FANCD2 (Chan et al, 2009), followed by the recruitment of TOPBP1 (Pedersen et al, 2015; Gallina et al, 2016; Leimbacher et al, 2019), which facilitates the assembly of additional factors (Bagge et al, 2025). Among these factors, SLX4 is a crucial scaffold protein that coordinates recruitment and activation of structure-specific nucleases such as MUS81-EME1 (Andersen et al, 2009; Fekairi et al, 2009; Muñoz et al, 2009; Svendsen et al, 2009; Castor et al, 2013; Garner et al, 2013; Wyatt et al, 2013), XPF-ERCC1 (Payliss et al, 2021) and SLX1 (Wyatt et al, 2017). In addition to nuclease activity, MiDAS also requires factors involved in DNA synthesis and repair, such as the strand-annealing protein RAD52 (Bhowmick et al, 2016), the POLD3 subunit of polymerase δ (Minocherhomji et al, 2015), and translesion synthesis polymerases Pol ζ and REV1 (Wu et al, 2023). However, the regulatory network governing mitotic responses to replication stress remains incompletely understood.

Cancerous Inhibitor of protein phosphatase 2 A (CIP2A) is an oncogenic factor initially characterized as an inhibitor of protein phosphatase 2 A (PP2A), involved in promoting tumor growth and proliferation (Junttila et al, 2007). Recently, CIP2A has been implicated in protecting chromosome integrity during mitosis (Adam et al, 2021; De Marco Zompit et al, 2022). CIP2A interacts with Topoisomerase II-binding protein 1 (TOPBP1) to form a complex required for tethering DNA double-strand breaks (DSBs) during mitosis in an MDC1-dependent manner (De Marco Zompit et al, 2022). CIP2A has also emerged as a synthetic lethal partner of BRCA1 and BRCA2, highlighting its potential as a therapeutic target in cancers harboring these mutations. However, the mechanistic basis of this interaction remains unclear (Adam et al, 2021).

Notably, cells deficient in BRCA2 spontaneously activate MiDAS, likely as a compensatory response to chronic replication stress (Feng and Jasin, 2017; Groelly et al, 2022). Given that CIP2A exhibits synthetic lethality with BRCA deficiency, and directly interacts with TOPBP1 in mitosis, we hypothesized that CIP2A contributes to mitotic responses to replication stress.

Here, we investigate the role of CIP2A in mitotic responses to replication stress under conditions of BRCA2 loss, ATR inhibition, and replication blockade. We demonstrate that CIP2A regulates the recruitment of the structure-specific endonuclease complex SLX4–MUS81-XPF (SMX complex), and that its loss leads to increased chromosomal instability under replication stress, manifesting as anaphase defects and micronuclei formation. We further show that CIP2A contributes to mitotic DNA synthesis (MiDAS) in a cell line-dependent manner. Importantly, SMX complex recruitment and MiDAS can be genetically separable processes. Altogether, we provide mechanistic insight into how mitotic replication stress is resolved to maintain genome stability.

## Results and discussion

### The CIP2A-TOPBP1 complex responds to replication stress in mitosis

**Figure 1:**
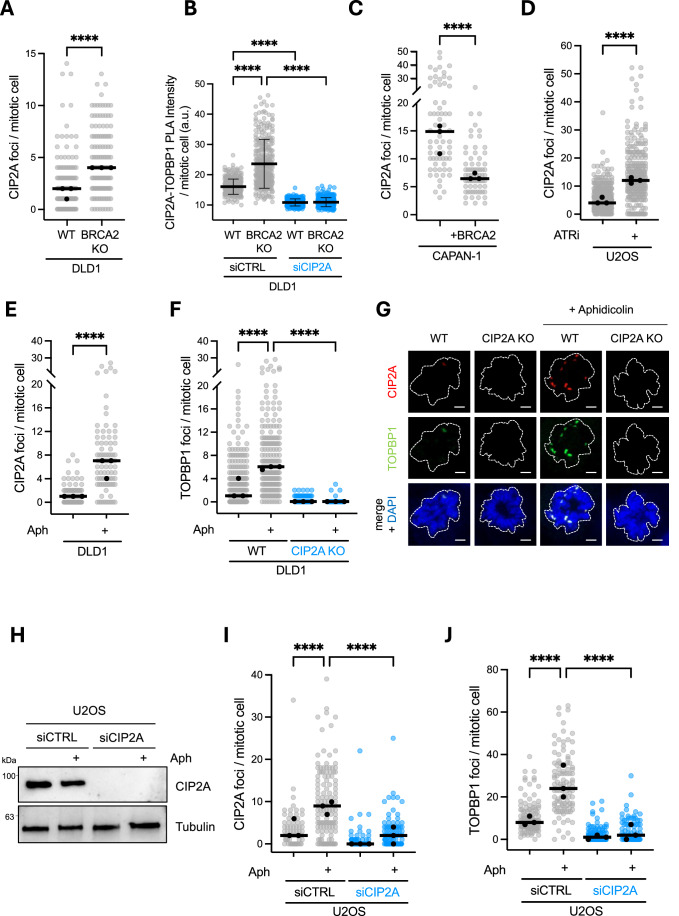
The CIP2A-TOPBP1 complex responds to replication stress in mitosis. (**A**) Dot plot showing number of CIP2A foci per mitotic cell in DLD1 WT and DLD1 BRCA2 KO. Replicate medians (black dots) and the pooled median (black bar) are indicated. *n* = 3 biological replicates. Statistical analysis: Mann–Whitney test. (**B**) Dot plot showing CIP2A-TOPBP1 mean PLA intensity per mitotic cell in DLD1 WT and DLD1 BRCA2 KO following transfection with control siRNA (siCTRL) or siCIP2A. Mean ± SD (black bar) is indicated. *n* = 3 biological replicates. Statistical analysis: Kruskal–Wallis test. (**C**) Dot plot showing number of CIP2A foci per mitotic cell in CAPAN-1 and CAPAN-1 complemented with wild-type BRCA2 ( + BRCA2). Replicate medians (black dots) and the pooled median (black bar) are indicated. *n* = 3 biological replicates. Statistical analysis: Mann–Whitney test. (**D**) Dot plot showing CIP2A foci per mitotic cell in U2OS treated with ATR inhibitor (ATRi). Cells are treated or not with 10 µM ATRi µM for 24 h. In the last 7 h of treatment, CDK1 inhibitor RO-3306 is added at 7 µM. Cells are then washed with PBS, released into fresh media and collected after 30 min. Replicate medians (black dots) and the pooled median (black bar) are indicated. *n *= 3 biological replicates. Statistical analysis: Mann–Whitney test. (**E**) Dot plot showing CIP2A foci per mitotic cell in DLD1 treated with aphidicolin (Aph). Cells are treated or not with 0.4 µM aphidicolin for 24 h. In the last 6 h of treatment, CDK1 inhibitor RO-3306 is added at 7 µM. Cells are then washed with PBS, released into fresh media and collected after 25 min. Replicate medians (black dots) and the pooled median (black bar) are indicated. *n* = 3 biological replicates. Statistical analysis: Mann–Whitney test. (**F**) Dot plot showing TOPBP1 foci per mitotic cell in DLD1 and DLD1 CIP2A KO (cl. 7) treated as in (**E**). Replicate medians (black dots) and the pooled median (black bar) are indicated. *n* = 3 biological replicates. Statistical analysis: Kruskal–Wallis test. (**G**) Representative confocal microscopy images of metaphase DLD1 cells showing CIP2A (red), TOPBP1 (green), and DAPI (blue). Images are maximum projections of Z-stacks. Cell outlines are indicated by dotted lines. Scale bar is 5 µm. (**H**) Western blot showing total protein levels of CIP2A in U2OS cells treated or not with aphidicolin following transfection with control siRNA (siCTRL) or siCIP2A. *n* = 2 biological replicates; a representative blot is shown. (**I**) Dot plot showing CIP2A foci per mitotic cell in U2OS treated with aphidicolin (Aph) following transfection with control siRNA (siCTRL) or siCIP2A. Cells are treated or not with 0.4 µM aphidicolin for 24 h. In the last 7 h of treatment, CDK1 inhibitor RO-3306 is added at 7 µM. Cells are then washed with PBS, released into fresh media and collected after 30 min. Replicate medians (black dots) and the pooled median (black bar) are indicated. *n* = 3 biological replicates. Statistical analysis: Kruskal–Wallis test. (**J**) Dot plot showing TOPBP1 foci per mitotic cell in U2OS treated with aphidicolin (Aph) as in (**I**) following transfection with control siRNA (siCTRL) or siCIP2A. Replicate medians (black dots) and the pooled median (black bar) are indicated. *n* = 3 biological replicates. Statistical analysis: Kruskal–Wallis test. For all the panels, data are presented with the following significance thresholds: ns (not significant), *P* < 0.05 (*), *P* < 0.01 (**), *P* < 0.001 (***), and *P* < 0.0001 (****). Source data are available online for this figure.

**Figure EV1:**
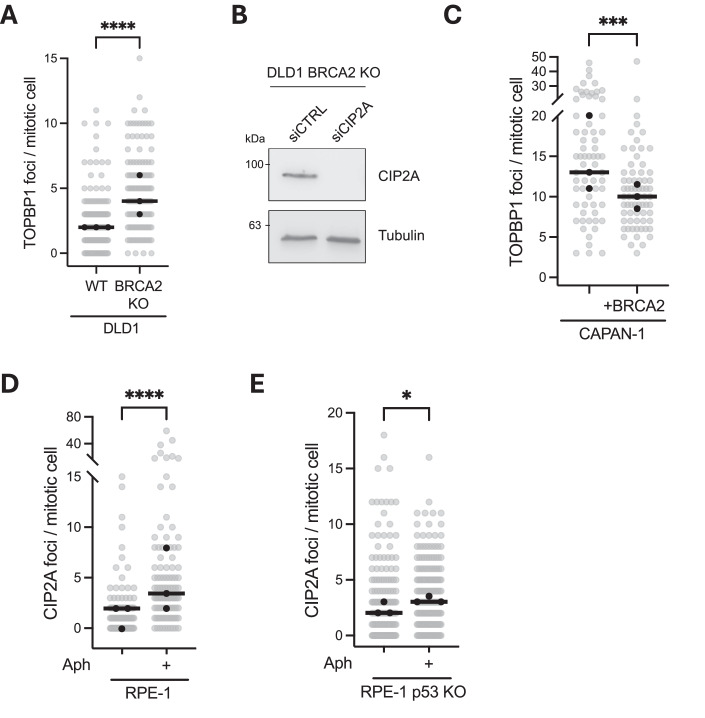
The CIP2A-TOPBP1 complex responds to replication stress across multiple cell lines. (**A**) Dot plot showing number of TOPBP1 foci per mitotic cell in DLD1 WT and DLD1 BRCA2 KO. Replicate medians (black dots) and the pooled median (black bar) are indicated. *n* = 3 biological replicates. Statistical analysis: Mann–Whitney test. (**B**) Western blot showing total protein levels of CIP2A in DLD1 BRCA2 KO cells following transfection with control siRNA (siCTRL) or siCIP2A. *n *= 2 biological replicates; a representative blot is shown. (**C**) Dot plot showing number of TOPBP1 foci per mitotic cell in CAPAN-1 and CAPAN-1 complemented with wild-type BRCA2 ( + BRCA2). Replicate medians (black dots) and the pooled median (black bar) are indicated. *n* = 3 biological replicates. Statistical analysis: Mann–Whitney test. (**D**) Dot plot showing CIP2A foci per mitotic cell in RPE-1 treated with aphidicolin (Aph). Cells are treated or not with 0.4 µM aphidicolin for 24 h. In the last 6 h of treatment, CDK1 inhibitor RO-3306 is added at 7 µM. Cells are then washed with PBS, released into fresh media and collected after 25 min. Replicate medians (black dots) and the pooled median (black bar) are indicated. *n* = 3 biological replicates. Statistical analysis: Mann–Whitney test. (**E**) Dot plot showing CIP2A foci per mitotic cell in RPE-1 p53 KO treated with aphidicolin (Aph). Cells are treated or not with 0.4 µM aphidicolin for 24 h. In the last 6 h of treatment, CDK1 inhibitor RO-3306 is added at 7 µM. Cells are then washed with PBS, released into fresh media and collected after 25 min. Replicate medians (black dots) and the pooled median (black bar) are indicated. *n* = 3 biological replicates. Statistical analysis: Mann–Whitney test. For all the panels, data are presented with the following significance thresholds: ns (not significant), *P* < 0.05 (*), *P *< 0.01 (**), *P* < 0.001 (***), and *P* < 0.0001 (****).

To investigate CIP2A’s role in response to replication stress, we first looked at its recruitment in the form of foci during mitosis under various replication stress conditions. Consistent with our previous findings (Adam et al, 2021), CIP2A and TOPBP1 foci were significantly increased in BRCA2 KO DLD1 mitotic cells (Figs. 1A and EV1A). In addition, the proximity of CIP2A with TOPBP1 was enhanced specifically in BRCA2 KO mitotic cells, as determined by proximity ligation assay (PLA; Figs. 1B and EV1B). Similar results were obtained in CAPAN-1 cells, which harbor a BRCA2 mutation, compared with BRCA2-complemented controls (Figs. 1C and EV1C).

Next, we induced replication stress by inhibiting ATR with VE-821 (ATRi) and found that CIP2A foci were increased in mitotic cells (Fig. 1D), suggesting that CIP2A not only responds to BRCA-deficiency-associated replication stress but also to ATRi-induced replication stress. Notably, CIP2A-deficient cells exhibit high sensitivity to ATR inhibitor treatment, but the underlying cause remains unknown (Hustedt et al, 2019).

Finally, we induced replication stress using low doses of aphidicolin, a replicative DNA polymerase inhibitor, to further assess whether CIP2A-TOPBP1 responds to stalled replication forks and under-replicated DNA in mitosis. Consistently, CIP2A and TOPBP1 were recruited in response to aphidicolin-induced replication stress in mitotic cells, as observed in both DLD1 (Fig. 1E–G) and U2OS cells (Fig. 1H–J). Similar recruitment was observed in RPE-1 cells (Fig. EV1D,E).

Importantly, TOPBP1 recruitment was abolished in the absence of CIP2A, either upon knockout in DLD1 cells or following siRNA-mediated depletion in U2OS cells, confirming the co-dependency of these two factors (Fig. 1F,G,J). (De Marco Zompit et al, 2022).

Collectively, these data demonstrate that the CIP2A-TOPBP1 complex responds robustly to diverse types of replication stress during mitosis, highlighting its broader role in the maintenance of genomic stability.

### CIP2A promotes recruitment of the SMX nuclease complex but not RAD52

It was previously proposed that upon replication stress, TOPBP1 maintains genome integrity in mitosis by controlling chromatin recruitment of the structure-specific nuclease scaffold protein SLX4 (Pedersen et al, 2015). SLX4 acts as a scaffold protein for MUS81-EME1 and XPF-ERCC1, which were shown to be recruited to fragile sites in mitosis (Naim et al, 2013; Ying et al, 2013). Since CIP2A acts as a regulator of TOPBP1 in mitosis, we tested whether CIP2A contributes to the recruitment of the SMX tri-nuclease complex in response to replication stress.

**Figure 2:**
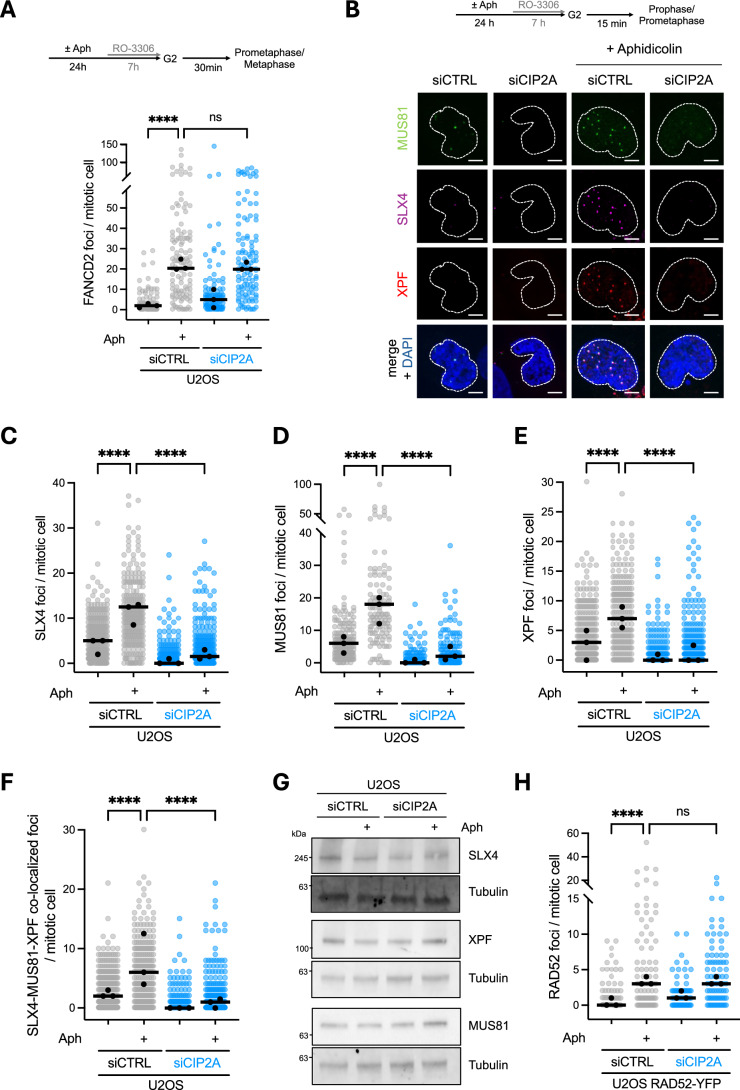
CIP2A mediates the recruitment of the SMX tri-nuclease complex to under-replicated DNA in mitosis. (**A**) Top, experimental scheme used to collect prometaphase and metaphase cells. Cells are treated or not with 0.4 µM aphidicolin (Aph) for 24 h. In the last 7 h of treatment, CDK1 inhibitor RO-3306 is added at 7 µM. Cells are then washed with PBS, released into fresh media and collected after 30 min. Bottom, dot plot showing number of FANCD2 foci per mitotic cell in U2OS. Replicate medians (black dots) and the pooled median (black bar) are indicated. *n* = 3 biological replicates. Statistical analysis: Kruskal–Wallis test. (**B**) Top, experimental scheme used to collect prometaphase cells. Cells are treated or not with 0.4 µM aphidicolin (Aph) for 24 h. In the last 7 h of treatment, CDK1 inhibitor RO-3306 is added at 7 µM. Cells are then washed with PBS, released into fresh media and collected after 15 min. Bottom, representative confocal microscopy images of prophase/prometaphase U2OS cells showing MUS81 (green), SLX4 (magenta), XPF (red), and DAPI (blue). Images are maximum projections of Z-stacks. Cell outlines are indicated by dotted lines. Scale bar is 5 µm. (**C**) Dot plot showing number of SLX4 foci per mitotic cell in U2OS treated as in (**B**) following transfection with control siRNA (siCTRL) or siCIP2A. Replicate medians (black dots) and the pooled median (black bar) are indicated. *n *= 3 biological replicates. Statistical analysis: Kruskal–Wallis test. (**D**) Dot plot showing number of MUS81 foci per mitotic cell in U2OS treated as in (**B**) following transfection with control siRNA (siCTRL) or siCIP2A. Replicate medians (black dots) and the pooled median (black bar) are indicated. *n *= 3 biological replicates. Statistical analysis: Kruskal–Wallis test. (**E**) Dot plot showing number of XPF foci per mitotic cell in U2OS treated as in (**B**) following transfection with control siRNA (siCTRL) or siCIP2A. Replicate medians (black dots) and the pooled median (black bar) are indicated. *n* = 3 biological replicates. Statistical analysis: Kruskal–Wallis test. (**F**) Dot plot showing number of SLX4–MUS81-XPF co-localizing foci per mitotic cell in U2OS treated as in (**B**) following transfection with control siRNA (siCTRL) or siCIP2A. Replicate medians (black dots) and the pooled median (black bar) are indicated. *n* = 3 biological replicates. Statistical analysis: Kruskal–Wallis test. (**G**) Western blot showing total protein levels of SLX4, MUS81, and XPF in U2OS cells treated or not with aphidicolin following transfection with control siRNA (siCTRL) or siCIP2A. *n* = 2 biological replicates; a representative blot is shown. (**H**) Dot plot showing number of RAD52 foci per mitotic cell in U2OS RAD52-YFP treated as in (**B**) following transfection with control siRNA (siCTRL) or siCIP2A. Replicate medians (black dots) and the pooled median (black bar) are indicated. *n* = 3 biological replicates. Statistical analysis: Kruskal–Wallis test. For all the panels, data are presented with the following significance thresholds: ns (not significant), *P* < 0.05 (*), *P* < 0.01 (**), *P* < 0.001 (***), and *P* < 0.0001 (****). Source data are available online for this figure.

We first examined FANCD2 recruitment, which marks under-replicated DNA and acts as an early upstream factor in the replication stress response (Chan et al, 2009). Cells were treated with a low dose of aphidicolin for a total of 24 h. During the last 7 h, RO-3306 was added to synchronize cells at the G2/M boundary. Cells were then released into mitosis and collected for immunofluorescence analysis. Notably, FANCD2 foci formation was unaffected by CIP2A depletion, indicating that CIP2A is not required for the initial recognition of under-replicated DNA (Fig. 2A). This further suggests that replication stress levels at mitotic entry are comparable between control and CIP2A-depleted cells.

Next, we analyzed the recruitment of SLX4, a scaffold protein that coordinates recruitment and activity of multiple structure-specific nucleases, including MUS81-EME1 and XPF-ERCC1, thereby facilitating their accumulation at sites of under-replicated DNA (Castor et al, 2013; Garner et al, 2013; Wyatt et al, 2013). Replication stress induction strongly increased SLX4 foci formation in mitotic control cells, consistent with its involvement in MiDAS; however, depletion of CIP2A significantly decreased SLX4 foci number (Fig. 2B,C).

In line with SLX4 function in recruiting MUS81-EME1, we observed a significant increase in MUS81 foci formation after aphidicolin treatment, which was almost completely abolished in absence of CIP2A (Fig. 2B,D). Similarly, XPF foci formation followed the same trend and was nearly abolished upon CIP2A depletion (Fig. 2B,E). Consistent with these observations, formation of SMX-positive foci containing SLX4, MUS81, and XPF was strongly reduced (Fig. 2B,F), despite unchanged total protein levels (Fig. 2G).

**Figure EV2:**
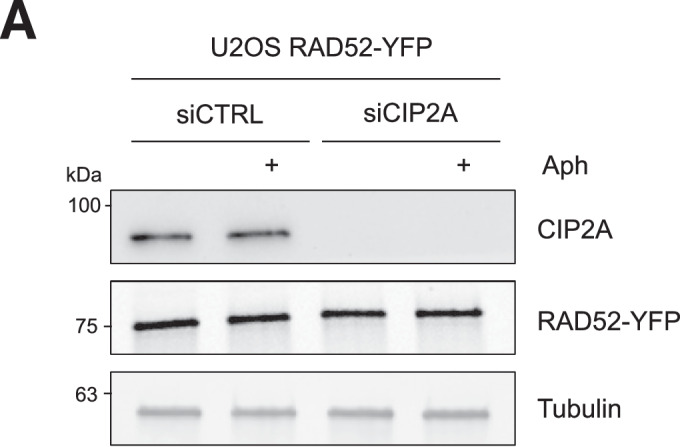
CIP2A silencing in U2OS RAD52-YFP cells. (**A**) Western blot showing total protein levels of CIP2A and RAD52-YFP in U2OS RAD52-YFP cells treated or not with aphidicolin following transfection with control siRNA (siCTRL) or siCIP2A.

Finally, we examined the recruitment of RAD52, which has also been implicated in the mitotic response to replication stress as a strand-annealing factor necessary to achieve BIR-like replication (Bhowmick et al, 2016). Unlike MUS81, XPF, and SLX4, RAD52 foci formation was unaffected by CIP2A depletion, suggesting that CIP2A selectively regulates SMX complex recruitment without affecting RAD52 binding (Figs.2H and EV2A).

Collectively, these findings demonstrate that CIP2A is required for the recruitment of SLX4, MUS81 and XPF to sites of under-replicated DNA during mitosis, while not affecting RAD52 recruitment.

### CIP2A contributes to MiDAS in a context-dependent manner

**Figure 3:**
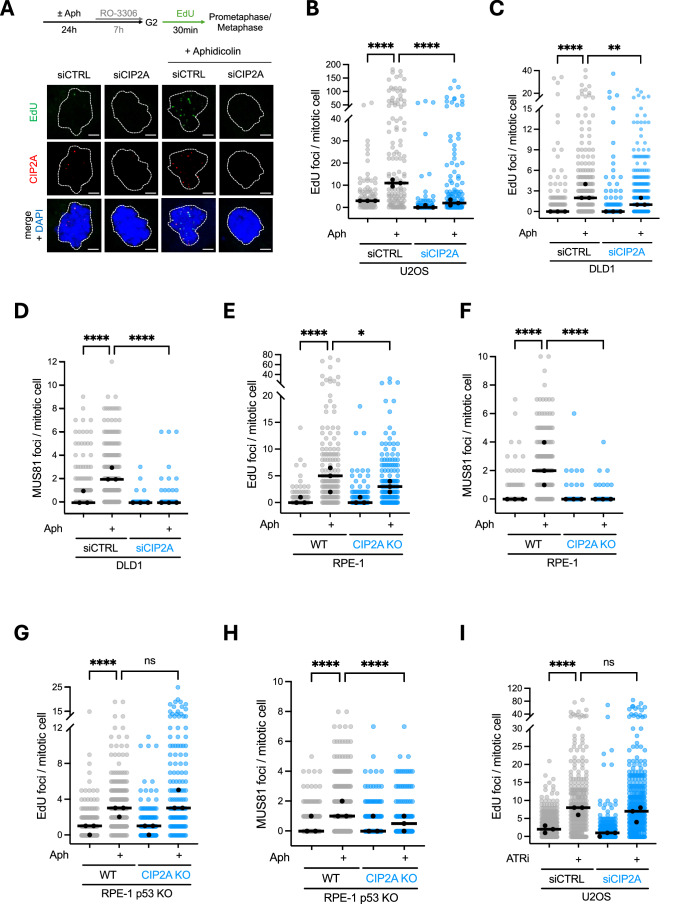
CIP2A involvement in MiDAS is cell line dependent. (**A**) Top, experimental scheme used to collect prometaphase and metaphase cells. Cells are treated or not with 0.4 µM aphidicolin (Aph) for 24 h. In the last 7 h of treatment, CDK1 inhibitor RO-3306 is added at 7 µM. Cells are then washed with PBS, released into fresh media containing EdU and collected after 30 min. Bottom, representative confocal microscopy images of prometaphase cells showing CIP2A (red), EdU (green), and DAPI (blue). Images are maximum projections of Z-stacks. Cell outlines are indicated by dotted lines. Scale bar is 5 µm. (**B**) Dot plot showing number of EdU foci per mitotic cell in U2OS treated as in (**A**) following transfection with control siRNA (siCTRL) or siCIP2A. Replicate medians (black dots) and the pooled median (black bar) are indicated. *n* = 3 biological replicates. Statistical analysis: Kruskal–Wallis test. (**C**) Dot plot showing number of EdU foci per mitotic cell in DLD1 treated as in (**A**) but collected after 25 min, following transfection with control siRNA (siCTRL) or siCIP2A. Replicate medians (black dots) and the pooled median (black bar) are indicated. *n* = 3 biological replicates. Statistical analysis: Kruskal–Wallis test. (**D**) Dot plot showing number of MUS81 foci per mitotic cell in DLD1 treated as in (**A**) but collected after 25 min, following transfection with control siRNA (siCTRL) or siCIP2A. Replicate medians (black dots) and the pooled median (black bar) are indicated. *n* = 3 biological replicates. Statistical analysis: Kruskal–Wallis test. (**E**) Dot plot showing number of EdU foci per mitotic cell in RPE-1 and RPE-1 CIP2A KO treated as in (**A**) but collected after 25 min. Replicate medians (black dots) and the pooled median (black bar) are indicated. *n* = 3 biological replicates. Statistical analysis: Kruskal–Wallis test. (**F**) Dot plot showing number of MUS81 foci per mitotic cell in RPE-1 and RPE-1 CIP2A KO treated as in (**A**) but collected after 25 min. Replicate medians (black dots) and the pooled median (black bar) are indicated. *n* = 3 biological replicates. Statistical analysis: Kruskal–Wallis test. (**G**) Dot plot showing number of EdU foci per mitotic cell in RPE-1 p53 KO and RPE-1 p53 KO CIP2A KO treated as in (**A**) but collected after 25 min. Replicate medians (black dots) and the pooled median (black bar) are indicated. *n* = 3 biological replicates. Statistical analysis: Kruskal–Wallis test. (**H**) Dot plot showing number of MUS81 foci per mitotic cell in RPE-1 p53 KO and RPE-1 p53 KO CIP2A KO treated as in (**A**) but collected after 25 min. Replicate medians (black dots) and the pooled median (black bar) are indicated. *n* = 3 biological replicates. Statistical analysis: Kruskal–Wallis test. (**I**) Dot plot showing number of EdU foci per mitotic cell in U2OS treated as in (**A**) but with 10 µM ATRi, following transfection with control siRNA (siCTRL) or siCIP2A. Replicate medians (black dots) and the pooled median (black bar) are indicated. *n* = 3 biological replicates. Statistical analysis: Kruskal–Wallis test. For all the panels, data are presented with the following significance thresholds: ns (not significant), *P* < 0.05 (*), *P* < 0.01 (**), *P* < 0.001 (***), and *P* < 0.0001 (****). Source data are available online for this figure.

We next examined whether CIP2A also contributes to mitotic DNA synthesis (MiDAS). Following aphidicolin-induced replication stress, U2OS cells displayed robust EdU incorporation in mitosis, indicative of MiDAS. CIP2A depletion significantly reduced mitotic EdU foci in this cell line (Fig. 3A,B), suggesting a role in promoting MiDAS under these conditions.

**Figure EV3:**
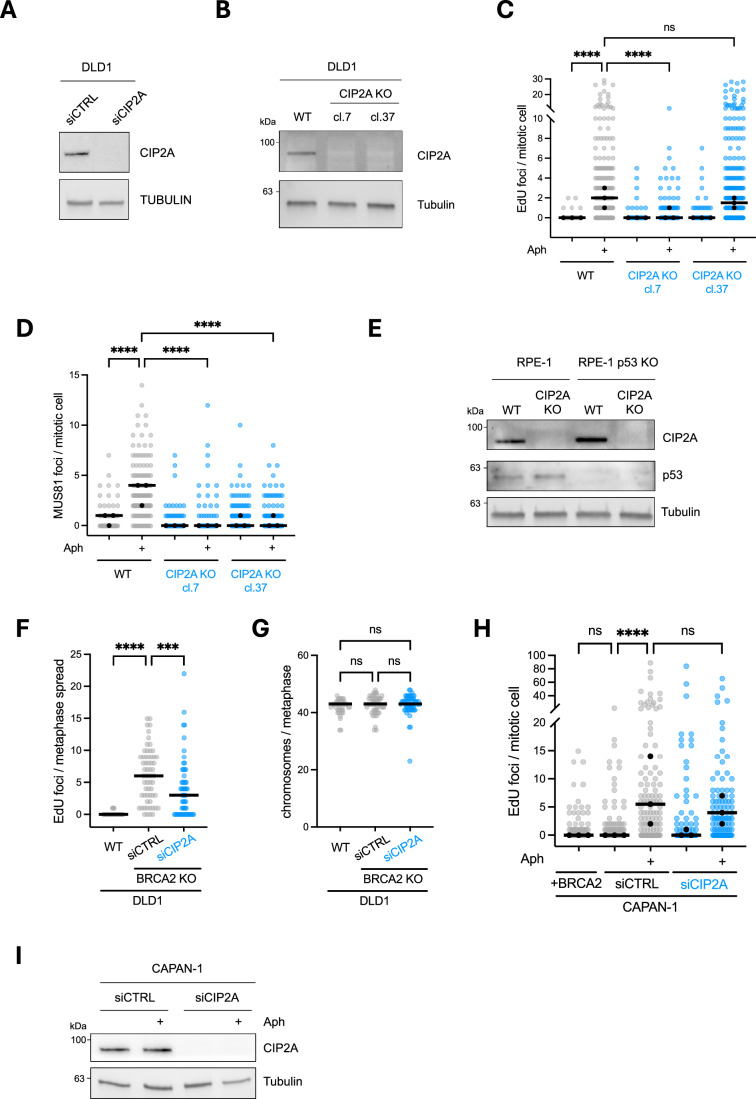
Additional analyses of CIP2A dependency for MiDAS across cell lines. (**A**) Western blot showing total protein levels of CIP2A in DLD1 cells following transfection with control siRNA (siCTRL) or siCIP2A. (**B**) Western blot showing total protein levels of CIP2A in DLD1 WT, CIP2A KO cl.7 and c. 37. (**C**) Dot plot showing number of EdU foci per mitotic cell in DLD1 WT, CIP2A KO cl.7 and c. 37 treated as in Fig. 3A but collected after 25 min. Replicate medians (black dots) and the pooled median (black bar) are indicated. *n* = 3 biological replicates. Statistical analysis: Kruskal–Wallis test. (**D**) Dot plot showing number of MUS81 foci per mitotic cell in DLD1 WT, CIP2A KO cl.7 and c. 37 treated as in Fig. 3A but collected after 25 min. Replicate medians (black dots) and the pooled median (black bar) are indicated. *n *= 3 biological replicates. Statistical analysis: Kruskal–Wallis test. (**E**) Western blot showing total protein levels of CIP2A and p53 in RPE-1, RPE-1 CIP2A KO, RPE-1 p53 KO, RPE-1 p53 KO CIP2A KO. (**F**) Dot plot showing number of EdU foci per metaphase spread in DLD1 WT and BRCA2 KO, following transfection with control siRNA (siCTRL) or siCIP2A. Cells were synchronized with RO-3306 for 4 h, then released in presence of Colcemid and EdU for 1 h and collected. Pooled median (black bar) indicated. *n* = 2 biological replicates. Statistical analysis: Kruskal–Wallis test. (**G**) Dot plot showing number of chromosomes per metaphase spread in DLD1 WT and BRCA2 KO, following transfection with control siRNA (siCTRL) or siCIP2A. Pooled median (black bar) indicated. *n* = 2 biological replicates. Statistical analysis: Kruskal–Wallis test. (**H**) Dot plot showing number of EdU foci per mitotic cell in CAPAN-1 + BRCA2 and CAPAN-1, following transfection with control siRNA (siCTRL) or siCIP2A, treated as in Fig. 3A. Replicate medians (black dots) and the pooled median (black bar) are indicated. *n* = 3 biological replicates. Statistical analysis: Kruskal–Wallis test. (**I**) Western blot showing total protein levels of CIP2A in CAPAN-1 cells treated or not with aphidicolin following transfection with control siRNA (siCTRL) or siCIP2A. *n* = 2 biological replicates; a representative blot is shown. For all the panels, data are presented with the following significance thresholds: ns (not significant), *P* < 0.05 (*), *P* < 0.01 (**), *P* < 0.001 (***), and *P* < 0.0001 (****).

However, this effect was not conserved across cell types. In DLD1 cells, CIP2A depletion by siRNA led to only a modest reduction of already low levels of MiDAS, despite a complete loss of MUS81 recruitment (Figs. 3C,D and EV3A). Two published DLD1 CIP2A knock-out clones (Adam et al, 2021) displayed distinct MiDAS phenotypes while consistently showing complete loss of MUS81 foci upon CIP2A knockout (Fig. EV3B–D). Similarly, RPE-1 cells exhibited low basal MiDAS levels that were only slightly affected by CIP2A loss despite complete loss of MUS81 foci (Figs. 3E,F and EV3E). MiDAS was largely independent of CIP2A in RPE-1 p53 knockout cells, consistent with a recent report (Fig. 3G,H) (de Haan et al, 2025).

Acute depletion of BRCA2 was shown to exhibit spontaneous low levels of MiDAS activity (Feng and Jasin, 2017; Groelly et al, 2022). In BRCA2 knock-out DLD1 cells, which display low but detectable MiDAS, CIP2A depletion resulted in a modest decrease in EdU incorporation (Fig. EV3F,G), consistent with a recent report (Martin et al, 2025). However, we did not observe increased spontaneous MiDAS in BRCA2-deficient CAPAN-1 cells compared to their wild-type BRCA2-complemented derivatives. MiDAS could be induced in CAPAN-1 cells though by low-dose aphidicolin, but this response was not dependent on CIP2A (Fig. EV3H,I). Finally, although ATR inhibition increased MiDAS levels in U2OS cells, CIP2A depletion did not affect EdU incorporation under these conditions (Fig. 3I).

Collectively, these results indicate that the contribution of CIP2A to MiDAS is cell-type dependent and generally modest compared with its robust role in SMX recruitment. These findings further suggest that SMX recruitment and MiDAS can be genetically separable processes.

### Disruption of the CIP2A-TOPBP1 interaction abrogates MUS81 recruitment but does not affect MiDAS

**Figure 4:**
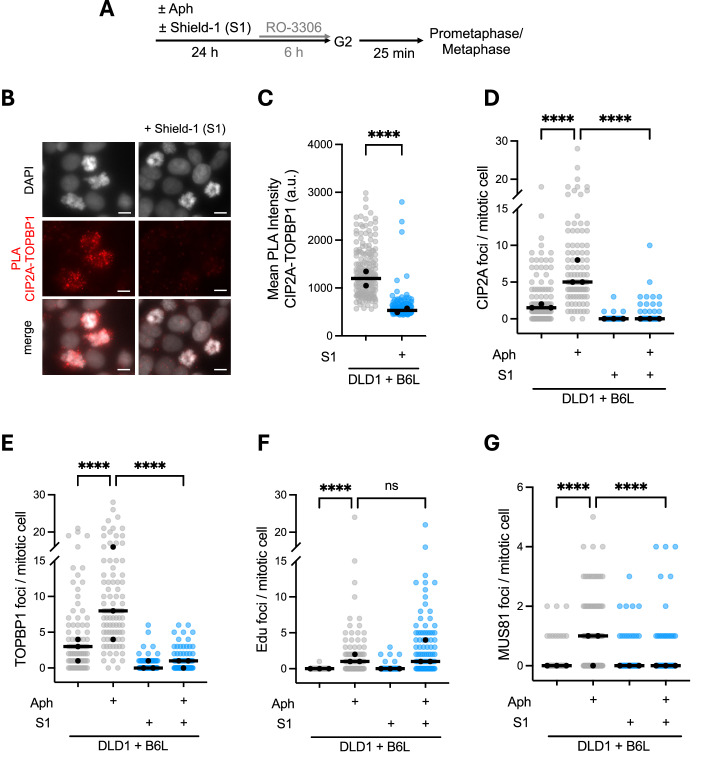
MUS81 recruitment, but not MiDAS, is affected by the disruption of CIP2A–TOPBP1 interaction. (**A**) Experimental scheme used to disrupt CIP2A–TOPBP1 interaction and collect prometaphase and metaphase cells. DLD1 B6L expressing cells are treated or not with 0.4 µM aphidicolin (Aph) and 1 µM Shield-1 (S1) for 24 h. In the last 6 h of treatment, CDK1 inhibitor RO-3306 is added at 7 µM. Cells are then washed with PBS, released into fresh media and collected after 25 min. (**B**) Representative confocal microscopy images of mitotic cells showing PLA CIP2A-TOPBP1 (red) and DAPI (white). Images are maximum projections of Z-stacks. Scale bar is 10 µm. (**C**) Dot plot showing mean CIP2A-TOPBP1 PLA intensity per mitotic cell in DLD1 WT B6L treated as in (**A**). Replicate medians (black dots) and the pooled median (black bar) are indicated. *n* = 2 biological replicates. Statistical analysis: Kruskal–Wallis test. (**D**) Dot plot showing number of CIP2A foci per mitotic cell in DLD1 WT B6L treated as in (**A**). Replicate medians (black dots) and the pooled median (black bar) are indicated. *n* = 3 biological replicates. Statistical analysis: Kruskal–Wallis test. (**E**) Dot plot showing number of TOPBP1 foci per mitotic cell in DLD1 WT B6L treated as in (**A**). Replicate medians (black dots) and the pooled median (black bar) are indicated. *n *= 3 biological replicates. Statistical analysis: Kruskal–Wallis test. (**F**) Dot plot showing number of EdU foci per mitotic cell in DLD1 WT B6L treated as in (**A**). Replicate medians (black dots) and the pooled median (black bar) are indicated. *n* = 3 biological replicates. Statistical analysis: Kruskal–Wallis test. (**G**) Dot plot showing number of MUS81 foci per mitotic cell in DLD1 WT B6L treated as in (**A**). Replicate medians (black dots) and the pooled median (black bar) are indicated. *n* = 3 biological replicates. Statistical analysis: Kruskal–Wallis test. For all the panels, data are presented with the following significance thresholds: ns (not significant), *P* < 0.05 (*), *P* < 0.01 (**), *P* < 0.001 (***), and *P* < 0.0001 (****). Source data are available online for this figure.

Our data suggest that MiDAS and SMX recruitment are not functionally linked, as CIP2A can be dispensable for MiDAS depending on the cellular context, while remaining essential for SMX recruitment (Figs. 2 and 3). To further test this, we asked whether disruption of the CIP2A-TOPBP1 interaction affects MiDAS activity and MUS81 foci formation. To this end, we used a previously described approach consisting of a DLD1 WT cell line expressing a fragment of TOPBP1, referred to as BRCT6-long (B6L, residues 756–1000) (Adam et al, 2021). This fragment is fused to an FKBP12-derived destabilization domain and can be stabilized by treatment with Shield-1 (S1), resulting in disruption of the TOPBP1-CIP2A interaction. To confirm the abrogation of the interaction between CIP2A and TOPBP1, we performed a proximity ligation assay (PLA) in cells treated with S1 and observed a decrease in the mean intensity of the PLA signal for CIP2A and TOPBP1 upon the disruption of the CIP2A-TOPBP1 interaction (Fig. 4A–C). Consistently, DLD1 WT B6L expressing cells exhibit a reduction in both CIP2A and TOPBP1 foci following the treatment with S1, independently of the aphidicolin treatment (Fig. 4D,E). We next assessed the impact of the abrogation of the CIP2A-TOPBP1 interaction on MiDAS, and we did not observe a significant reduction in EdU foci in aphidicolin-stressed cells in which the expression of the B6L fragment was induced (Fig. 4F). However, MUS81 foci formation was reduced thus providing additional confirmation that MUS81 recruitment and MiDAS do not strictly correlate (Fig. 4G).

### CIP2A loss leads to chromosomal instability upon replication stress

To investigate the consequences of CIP2A loss under replication stress, we analyzed genome instability markers following aphidicolin treatment. Specifically, we examined anaphase defects and micronuclei formation, two hallmarks of chromosomal missegregation and incomplete DNA replication.

**Figure 5:**
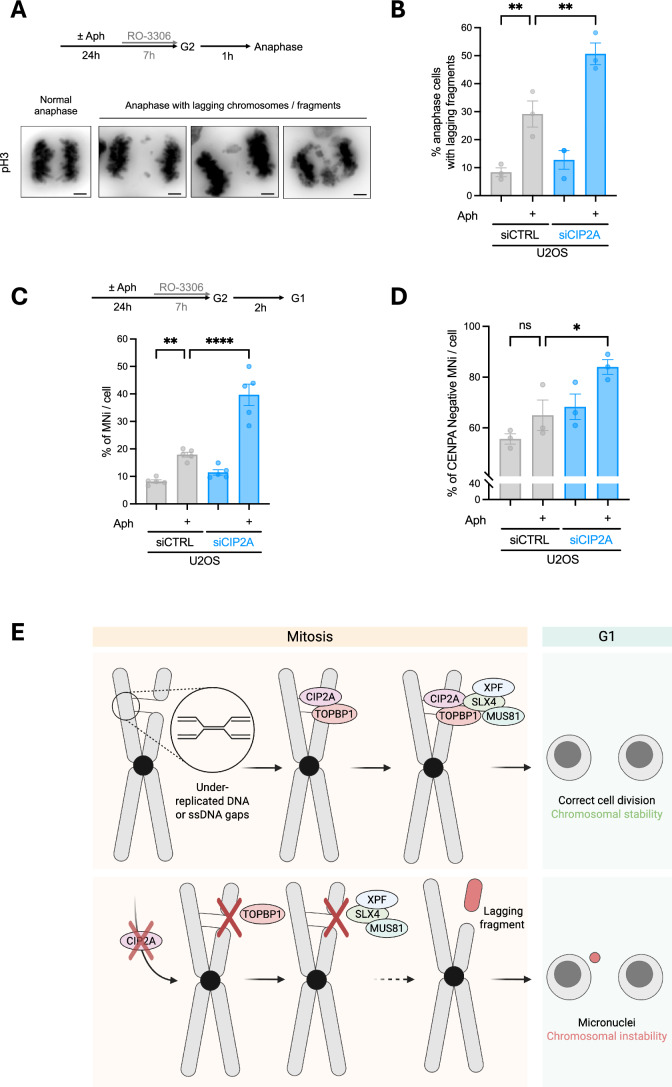
CIP2A loss is associated with chromosomal instability in response to replication stress. (**A**) Top, experimental scheme used to collect anaphase cells. Cells are treated or not with 0.4 µM aphidicolin (Aph) for 24 h. In the last 7 h of treatment, CDK1 inhibitor RO-3306 is added at 7 µM. Cells are then washed with PBS, released into fresh media and collected after 1 h. Bottom, representative examples of anaphase cells with lagging chromosomes or chromosome fragments, stained with pH3. Scale bar is 5 µm. (**B**) Bar plot showing the mean ± SEM of anaphase U2OS cells displaying lagging chromosomes or chromosome fragments treated as in A) following transfection with control siRNA (siCTRL) or siCIP2A. Statistical analysis: one-way ANOVA. *n* = 3 biological replicates shown as dots. (**C**) Top, experimental scheme used to collect G1 cells. Cells are treated or not with 0.4 µM aphidicolin (Aph) for 24 h. In the last 7 h of treatment, CDK1 inhibitor RO-3306 is added at 7 µM. Cells are then washed with PBS, released into fresh media and collected after 2 h. Bottom, bar plot showing the mean ± SEM of micronuclei (MNi) per G1 U2OS cell, following transfection with control siRNA (siCTRL) or siCIP2A. Statistical analysis: one-way ANOVA. *n* = 5 biological replicates shown as dots. (**D**) Bar plot showing the mean ± SEM of CENP-A Negative micronuclei (MNi) per G1 U2OS cell treated as in (**C**) following transfection with control siRNA (siCTRL) or siCIP2A. Statistical analysis: one-way ANOVA. *n* = 3 biological replicates shown as dots. (**E**) Proposed model for the role of CIP2A in the mitotic response to replication stress. Replication stress generates regions of under-replicated DNA and ssDNA gaps that persist into mitosis and trigger recruitment of the CIP2A-TOPBP1 complex. This complex promotes the recruitment of the SMX complex, including SLX4, MUS81, and XPF, facilitating the processing of replication intermediates before chromosome segregation. In the absence of CIP2A, recruitment of TOPBP1 and the SMX complex is impaired. Under replication stress, this leads to lagging chromosome fragments and micronuclei formation. Whether the failure to recruit the SMX complex directly causes this chromosomal instability remains to be determined. Created in https://BioRender.com. For all the panels, data are presented with the following significance thresholds: ns (not significant), *P* < 0.05 (*), *P* < 0.01 (**), *P* < 0.001 (***), and *P* < 0.0001 (****). Source data are available online for this figure.

We first assessed lagging chromosome fragments in anaphase cells, which were collected 1 h after release from G2/M synchronization. Control cells exhibited an increased frequency of lagging fragments upon treatment with a low dose of aphidicolin. However, in CIP2A-depleted cells, aphidicolin treatment led to a further increase in anaphase cells displaying lagging DNA fragments, indicating a failure to properly resolve DNA lesions caused by replication stress before chromosome segregation (Fig. 5A,B).

Next, we examined micronuclei formation in G1 cells, which were collected 2 h post-release from RO-3306 synchronization. CIP2A-depleted cells displayed a higher frequency of micronuclei compared to controls, suggesting that unresolved replication intermediates persist into the next cell cycle (Fig. 5C). Finally, to determine whether these micronuclei resulted from broken chromosome fragments rather than entire mis-segregated chromosomes, we performed CENP-A staining. CENP-A-negative micronuclei were significantly enriched in CIP2A-depleted cells, confirming that these structures originated from acentric chromosome fragments (Fig. 5D).

Altogether, these results demonstrate that CIP2A is a key regulator of the mitotic response to replication stress. CIP2A is required for the recruitment of TOPBP1 to sites of under-replicated DNA during mitosis and together they promote the recruitment of the SMX complex, thereby facilitating the processing of replication intermediates before chromosome segregation. CIP2A depletion increases chromosomal instability under replication stress, as evidenced by elevated anaphase lagging chromosomes and micronuclei formation. However, the causal link between defective SMX recruitment and these phenotypes remains to be established (Fig. 5E). These findings support CIP2A as a potential therapeutic target in cancers characterized by elevated replication stress, including BRCA-deficient tumors.

## Methods

**Table:** Reagents and Tools Table

Reagent/resource	Reference or source	Identifier or catalog number
**Experimental models**		
U-2 OS (U2OS)	ATCC	HTB-96
DLD-1 (DLD1)	ATCC	CCL-221
DLD1 CIP2A KO clone 7	Dan Durocher	Adam et al, 2021
DLD1 CIP2A KO clone 37	Dan Durocher	Adam et al, 2021
DLD1 + TOPBP1 BRCT6 FKBP12 (B6L)	Dan Durocher	Adam et al, 2021
DLD1 BRCA2 KO	ATCC	CVCL_HD57
hTERT RPE-1 (RPE-1)	ATCC	CRL-4000
RPE-1 CIP2A KO	This lab	De Marco Zompit et al, 2022
RPE-1 p53 KO	Dan Durocher	Adam et al, 2021
RPE-1 p53 KO CIP2A KO	Dan Durocher	Adam et al, 2021
U2OS RAD52-YFP	Jiri Lukas	Bekker-Jensen et al, 2006
CAPAN-1	Simon Powell	Xia et al, 2001
CAPAN-1 + BRCA2	Simon Powell	Xia et al, 2001
**Recombinant DNA**		
**Antibodies**		
Anti-CIP2A	Santa Cruz	sc-80659, RRID:AB_1121640
Anti-TOPBP1	Millipore	ABE1463,
Anti-CENPA	Abcam	ab13939, RRID:AB_300766
Anti-pSer10 Histone H3	Millipore	06-570, RRID:AB_310177
Anti-MUS81	Santa Cruz	sc-53382, RRID:AB_2147138
Anti-FANCD2	Novus Biologicals	NB100-182, RRID:AB_10002867
Anti-SLX4	John Rouse	Wilson et al, 2013
Anti-BTBD12 (SLX4)	Bethyl	A302-207A, RRID:AB_1850156
Anti-XPF	Abcam	ab76948, RRID:AB_1524575
Anti-RAD52	Santa Cruz	sc-365341, RRID:AB_10851346
Anti-p53	Abcam	ab32389, RRID:AB_776981
Anti-Tubulin	Bio-Rad	12004166
GFP-Booster Alexa Fluor® 488	Proteintech	gb2AF488-10
**Oligonucleotides and other sequence-based reagents**		
siCTRL	Microsynth AG	UGGUUUACAUGUCGACUAA-dTdT
siCIP2A	Human KIAA1524 siRNA SMARTpool	L-014135-01-0005
**Chemicals, enzymes and other reagents**		
Aphidicolin	MedChem Express	HY-N6733
RO-3306	SelleckChem	S7747
Karyomax Colcemid	Gibco	1521201
VE-821 ATR inhibitor	SelleckChem	S8007
Shield-1	Takara Bio	632189
Click-iT™ EdU Cell Proliferation Kit	Thermo Fisher	C10337
Duolink® PLA Reagents	Sigma-Aldrich	DUO92001, DUO92005, DUO92008, DUO82049
**Software**		
CellProfiler 4.2.8	www.cellprofiler.org	Stirling et al, 2021
GraphPad Prism 11.0.0 (93)	www.graphpad.com	
Fiji	https://imagej.net/software/fiji/	Schindelin et al, 2012

### Cell lines

All cells were cultured at 37 °C in a humidified incubator with 5% CO_2_. DLD1 cells were maintained in RPMI-1640 medium supplemented with 10% fetal bovine serum (FBS) and 1% penicillin-streptomycin. DLD1 BRCA2 knockout (KO) cells were obtained from ATCC (HTB-96). DLD1 CIP2A KO clones 7 and 37 and DLD1 B6L expressing cells were a generous gift from Dan Durocher (Adam et al, 2021). U2OS and RPE-1 cells were cultured in DMEM supplemented with 10% FBS and 1% penicillin-streptomycin. The U2OS YFP-RAD52 expressing cell line was a gift from Jiri Lukas (Bekker-Jensen et al, 2006). RPE-1 CIP2A KO cells were previously generated in the lab (De Marco Zompit et al, 2022). RPE-1 p53 KO and RPE-1 p53 KO CIP2A KO cells were a gift from Dan Durocher (Adam et al, 2021). CAPAN-1 and CAPAN-1 + WT BRCA2 cells were a gift from Simon Powell (Xia et al, 2001). All cell lines were regularly tested for mycoplasma contamination.

### Gene silencing with RNA interference

Transient gene depletion was performed using Lipofectamine™ RNAiMAX Transfection Reagent (Invitrogen, 13778150), according to the manufacturer’s instructions. The following siRNAs are used at a final concentration of 20 nM: ON-TARGETplus Human KIAA1524 (57650) siRNA SMARTpool L-014135-01-0005 for CIP2A. The control siRNA (siCTRL, UGGUUUACAUGUCGACUAA-dTdT) is obtained from Microsynth AG. Experiments were performed at 48-72 h post-transfection.

### Drugs and treatments

The following compounds were utilized in this study at the following concentration, unless otherwise indicated in the corresponding figure legends: 0.4 μM aphidicolin (MedChem Express, HY-N6733), 7 μM RO-3306 (SelleckChem, S7747), 0.1 μg/mL Karyomax Colcemid (Gibco, 1521201), 10 μM VE-821 ATR inhibitor (SelleckChem, S8007), 1 μM Shield-1 (Takara Bio, 632189). To enrich for mitotic cells, cultures were treated with the indicated drug for a total of 24 h. During the final 6–7 h of treatment, 7 μM RO-3306 was added to synchronize cells at the G2/M boundary. Cells were then washed three times with warm PBS for a total of 5 min and released into mitosis in fresh, pre-warmed medium. Cells were collected and fixed according to the experimental scheme. The exact timing of mitotic collection was optimized for each cell line and is reported in the corresponding figure legends. As a general reference, prophase cells peak at 10–15 min after release; prometaphase/metaphase cells at 25–30 min; and anaphase cells at 1 h post-release.

### Immunofluorescence

Cells were grown on glass coverslips and fixed with 10% formalin (corresponding to 4% paraformaldehyde) for 15 min at room temperature (RT) or with ice-cold methanol for 15 min on ice. For SLX4 staining, pre-extraction with cold CSK buffer (100 mM PIPES pH 7, 100 mM NaCl, 300 mM sucrose, 3 mM MgCl_2_) supplemented with 0.5% Triton-X-100 was performed for 8 min prior to fixation. For standard immunofluorescence staining, cells were permeabilized with 0.5% Triton-X-100 in PBS for 15 min, then blocked with 10% FBS for 1 h, followed by overnight incubation with primary antibodies diluted in 5% FBS. After three PBS washes, cells were incubated with Alexa Fluor-conjugated secondary antibodies (Thermo Fisher Scientific) at a 1:1000 dilution in 5% FBS for 1 h. For XPF and SLX4 staining, 0.1% Triton-X-100 was added during primary and secondary antibodies incubations. Following three additional PBS washes, coverslips were mounted on glass microscopy slides using VECTASHIELD® PLUS Antifade Mounting Medium containing 0.5 μg/mL DAPI (Vector Laboratories).

For MiDAS visualization, cells were permeabilized and blocked in 0.5% Triton-X-100 with 3% BSA for 30 min. The Click-iT™ reaction (Thermo Fisher, Cat. No. C10337) was performed according to the manufacturer’s instructions, but using undiluted 10X additive as described (Garribba et al, 2018), for a total of 45 min. After a single wash with 3% BSA, primary antibodies were incubated overnight in 1% BSA, followed by the standard immunofluorescence protocol.

Images were acquired using a Zeiss LSM 900 confocal microscope with a 63x oil immersion objective and an Andor BC43 confocal microscope with a 60x oil immersion objective. Mitotic cells were manually selected based solely on the DAPI channel, based on signal intensity and morphology. On average, 30–80 mitotic cells were imaged per condition using sequential scanning mode across a 6–8 μm z-stack with 8–12 optical sections.

### Image analysis

Microscopic images were analyzed in Fiji and CellProfiler 4.0 (Schindelin et al, 2012; Stirling et al, 2021). Total signal intensity and the number of foci per cell were quantified using customized pipelines developed in CellProfiler. All images were processed in batch using the same automated pipeline. Nuclei were segmented using the intensity-based Primary Object Detection module on the DAPI channel. Foci were identified either using the Primary Object Detection module or via the Find Maxima module. The CellProfiler pipelines used in this study are available upon request. Micronuclei and anaphase cells with lagging chromosomes were quantified manually.

### In-situ proximity ligation assay (PLA)

Cells were seeded on glass coverslips in 24-well plate at a density of 8 × 10^4^ cells per well and treated as indicated in the corresponding figure legends. Cells were synchronized in G2/M with 7 μM RO-3306 (Merck, 217699) 6 h before the end of the treatment. Mitotic release was performed by washing cells 3× with pre-warmed PBS and by incubating the cells for 25 min in fresh medium. Coverslips were washed with cold PBS, fixed with ice-cold methanol for 10 min on ice and washed 3× with PBS. In-situ proximity ligation assay (PLA) was performed using Duolink^®^ PLA Reagents (Sigma-Aldrich, DUO92001, DUO92005, DUO92008, DUO82049) following the manufacturer’s protocol. Cells were blocked for 1 h at 37 °C, followed by staining with anti-CIP2A (Mouse, Santa Cruz, sc-80659, 1:800) and anti-TOPBP1 (Rabbit, Millipore, ABE1463, 1:300) antibodies overnight at 4 °C. Cells were then washed 2× with Wash Buffer A for 5 min and incubated with PLUS and MINUS PLA probes for 1 h at 37 °C. Cells were washed 2× with Wash Buffer A for 5 min. Ligation and polymerization steps were performed following the manufacturer’s instructions. Cells were washed 2 × 10 min with Wash Buffer B, followed by a final wash with 0.01× Wash Buffer B. Coverslips were mounted on glass microscopy slides with VECTASHIELD^®^ PLUS Antifade Mounting Medium with DAPI. Quantification was performed with ImageJ, following manual selection of mitotic cells.

### Metaphase spreads

Cells were grown to 90% confluence on a 6-cm plate and were either treated or untreated with aphidicolin (0.4 μM) for 24 h and synchronized with RO-3306 (7 μM) for 4 h. Cells were washed 3× with pre-warmed PBS and released into EdU (40 μM) and KaryoMax Colcemid (0.1 μg/mL) for the last 40 min. Cells were trypsinized and transferred to a 15 ml Falcon™ tube, centrifuged at 176 × *g* for 5 min and carefully resuspended in 2.5 ml of pre-warmed hypotonic buffer (15% FBS, 75 mM KCl) with intermittent agitation and incubated for 30 min at 37 °C. Cells were again pelleted at 176 × *g* for 5 min, the supernatant was discarded, and the cell pellet was resuspended in 100 μl of hypotonic buffer. Cells were fixed by adding drop-wise 2.5 ml of ice-cold MeOH:AcOH 3:1 while slowly vortexing followed by 40 min on ice. After centrifugation at 176 × *g* for 5 min at 4 °C, supernatant was discarded, and cells were resuspended in the remaining 70 μl of the fixation buffer. A total of 20 μl of the cell suspension was then dropped at a 45° angle onto a wet glass slide and air-dried overnight.

Metaphases were stained using the Click-iT EdU Cell Proliferation Kit for Imaging, Alexa Fluor 488 dye (Thermo Fisher, Cat. No. C10337). Firstly, slides were fixed with 4% formalin for 12 min, washed 3 × 5 min with PBS and blocked with 3% BSA for 30 min at RT. After permeabilization in 0.5% Triton-X in PBS for 20 min, slides were washed with PBS and a Click-iT reaction was performed according to the protocol provided by the supplier. Finally, slides were washed 3 ×5 min with 3% BSA in 0.5% Triton-X/PBS and metaphases were stained using VECTASHIELD® PLUS antifade mounting medium with DAPI and covered with a glass coverslip. Spreads were imaged using a Zeiss LSM 900 widefield microscope with a 40x water immersion objective. On average, 30–50 metaphase spreads were imaged per condition. Number of chromosomes per spread and EdU Foci were counted manually using ImageJ.

### Western blot

Cells were processed for western blot as described previously (Meroni et al, 2022). Briefly, cells were collected and resuspended in lysis buffer (50 mM tris-HCl pH 7.5, 20 mM NaCl, 1 mM MgCl2, 0.1% SDS) for 20 min on ice. Samples were sonicated for 5 sec, clarified, and the total protein concentration was measured using a Pierce BCA protein assay kit (23227, Thermo Fisher Scientific) according to the manufacturer’s instructions. Samples were mixed with 4× Laemmli Buffer (Bio-Rad, 1610747) and loaded on 4–20% Mini-PROTEAN TGX Stain-free Precast Gels (Bio-Rad, 4568094). Gels were transferred to 0.2 μm nitrocellulose membranes using the Trans-Blot® Turbo™ Transfer system (Bio-Rad, 1704158). Blots were blocked with 2.5% milk in TBS-Tween and incubated overnight at 4 °C with primary antibodies. After washes and secondary antibody incubation, blots were developed either using chemiluminescence or infra-red detection using on a ChemiDoc MP Imaging System (Bio-Rad).

### Antibodies

The following primary antibodies were used for immunofluorescence (IF) and/or western blot (WB) at the indicated dilutions: anti-CIP2A (Mouse, Santa Cruz, sc-80659, IF 1/800, WB 1/200-1000), anti-TOPBP1 (Rabbit, Millipore, ABE1463, IF 1/250), anti-CENPA (Mouse, Abcam, ab13939, IF 1/500), anti-pSer10 Histone H3 (Rabbit, Millipore, 06-570, IF 1/1000), anti-MUS81 (Mouse, Santa Cruz, sc-53382, IF 1/250, WB 1/500), anti-FANCD2 (Rabbit, Novus Biologicals, NB100-182, IF 1/200), anti-SLX4 (Sheep, generous gift from John Rouse (Wilson et al, 2013), IF 1/100), anti-SLX4 (Rabbit, Bethyl, A302-207A, WB 1/1000), anti-XPF (Rabbit, Abcam, ab76948, IF 1/100, WB 1/1000), anti-RAD52 (Mouse, Santa Cruz, sc-365341, WB 1/500), anti-p53 (Rabbit, Abcam, ab32389, WB 1/5000), anti-Tubulin (anti-Tubulin hFAB Rhodamine antibody Bio-Rad 12004166, 1/5000). For YFP-tagged RAD52, coverslips were incubated with the ChromoTek GFP-Booster Alexa Fluor® 488 (Proteintech, gb2AF488-10, 1/500) for 1 h at RT.

### Statistical analysis

Statistical analyses were performed using GraphPad Prism (version 11.0.0). The specific statistical test applied is indicated in the corresponding figure legends. Data are presented with the following significance thresholds: ns (not significant), *P* < 0.05 (*), *P* < 0.01 (**), *P* < 0.001 (***), and *P *< 0.0001 (****). No statistical methods were used to predetermine sample size, and no inclusion or exclusion criteria were applied.

For single-cell measurements (e.g., number of foci), data distributions were assessed for normality (Shapiro-Wilk test) and did not follow a normal distribution; therefore, non-parametric tests were applied. Graphs are shown as SuperPlots (Lord et al, 2020): individual data points are displayed as scatter plots, while the median of each independent biological replicate is indicated separately. The line represents the median across the three independent biological experiments. For population-based analyses (e.g., percentage of micronuclei), data are presented as mean values, with the mean of each independent biological replicate shown. The number of biological replicates is specified in each corresponding figure legend (*n* = x).

## Supplementary information

Peer Review File

Source data Fig. 1

Source data Fig. 2

Source data Fig. 3

Source data Fig. 4

Source data Fig. 5

Expanded View Figures

## Data Availability

This study includes no data deposited in external repositories. The source data of this paper are collected in the following database record: biostudies:S-SCDT-10_1038-S44319-026-00807-3.
