# Supplementary material for: CIP2A recruits SLX4-MUS81-XPF in mitosis and protects against replication stress
Source: EMBO Rep. 2026 May 26;27(13):3585–603. doi: 10.1038/s44319-026-00807-3 (PMC13354792; doi:10.1038/s44319-026-00807-3)
Supplement: Supplementary file 2 — Source data Fig. 1 [file 44319_2026_807_MOESM2_ESM.zip › Figure 1/1H/README 1H.rtf]

Loading order:	1)	marker	2)	U2OS siCTRL UT	3)	U2OS siCTRL APH	4)	U2OS siCIP2A UT	5)	U2OS siCIP2A APH
