# Supplementary figures and images for: CIP2A recruits SLX4-MUS81-XPF in mitosis and protects against replication stress
Source: EMBO Rep. 2026 May 26;27(13):3585–603. doi: 10.1038/s44319-026-00807-3 (PMC13354792; doi:10.1038/s44319-026-00807-3)

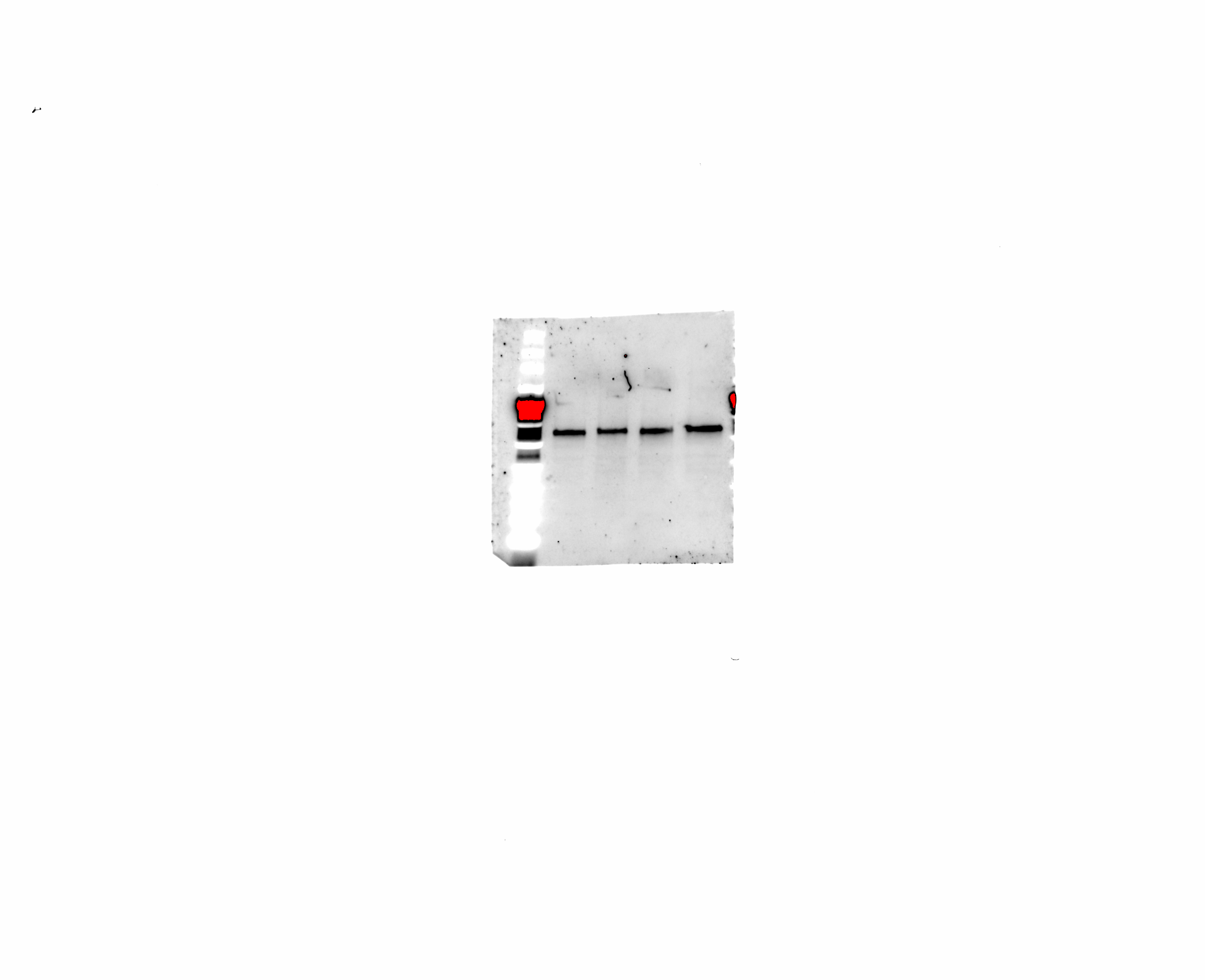

Supplement: Supplementary file 2 — Source data Fig. 1 [file 44319_2026_807_MOESM2_ESM.zip › Figure 1/1H/CIP2A TUBULIN.tif]

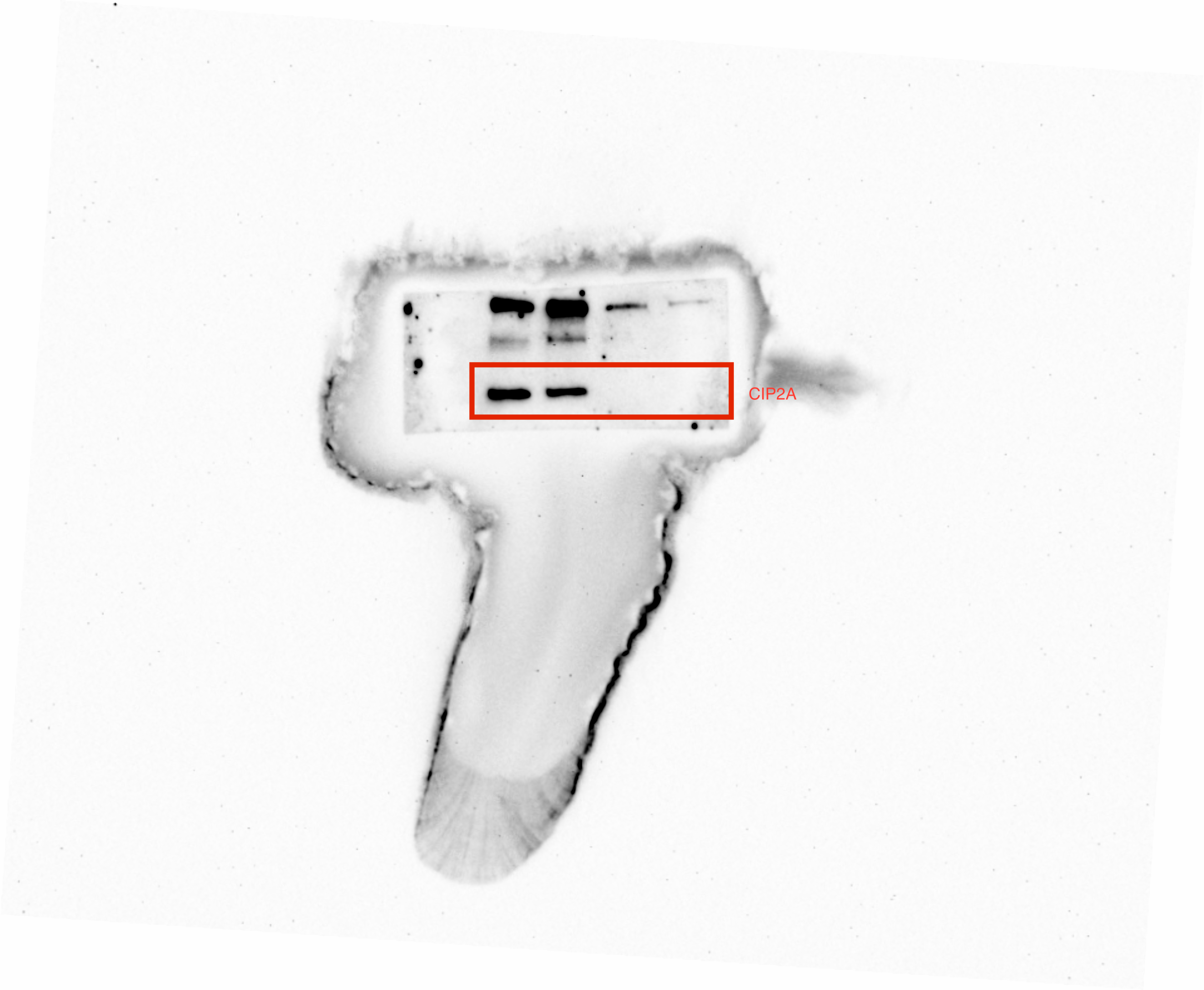

Supplement: Supplementary file 2 — Source data Fig. 1 [file 44319_2026_807_MOESM2_ESM.zip › Figure 1/1H/CIP2A.tif]

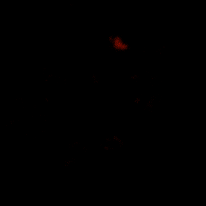

Supplement: Supplementary file 2 — Source data Fig. 1 [file 44319_2026_807_MOESM2_ESM.zip › Figure 1/1G/C3-MAX_DLD1_WT_untreated (10).tif]

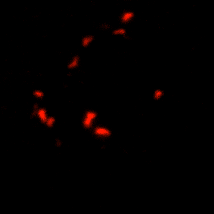

Supplement: Supplementary file 2 — Source data Fig. 1 [file 44319_2026_807_MOESM2_ESM.zip › Figure 1/1G/C3-MAX_DLD1_WT_APH (4).tif]

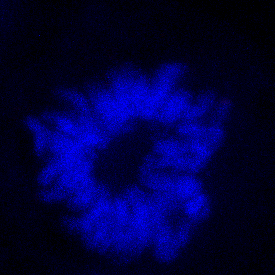

Supplement: Supplementary file 2 — Source data Fig. 1 [file 44319_2026_807_MOESM2_ESM.zip › Figure 1/1G/MAX_DLD1_CIP2A KO #7_untrated (8).tif (RGB).tif]

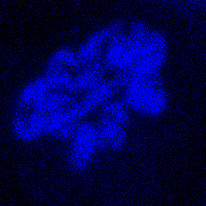

Supplement: Supplementary file 2 — Source data Fig. 1 [file 44319_2026_807_MOESM2_ESM.zip › Figure 1/1G/C2-MAX_DLD1_WT_untreated (10).tif]

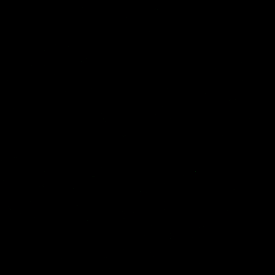

Supplement: Supplementary file 2 — Source data Fig. 1 [file 44319_2026_807_MOESM2_ESM.zip › Figure 1/1G/C1-MAX_DLD1_CIP2A KO #7_untrated (8).tif]

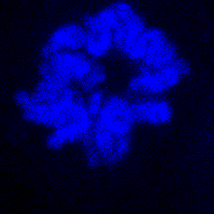

Supplement: Supplementary file 2 — Source data Fig. 1 [file 44319_2026_807_MOESM2_ESM.zip › Figure 1/1G/C2-MAX_DLD1_WT_APH (4).tif]

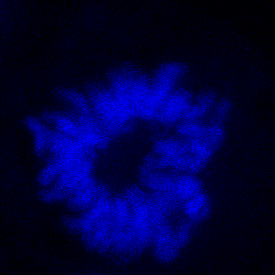

Supplement: Supplementary file 2 — Source data Fig. 1 [file 44319_2026_807_MOESM2_ESM.zip › Figure 1/1G/C2-MAX_DLD1_CIP2A KO #7_untrated (8).tif]

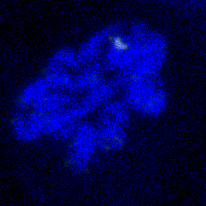

Supplement: Supplementary file 2 — Source data Fig. 1 [file 44319_2026_807_MOESM2_ESM.zip › Figure 1/1G/MAX_DLD1_WT_untreated (10).tif (RGB).tif]

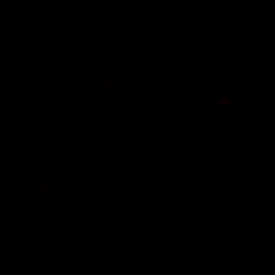

Supplement: Supplementary file 2 — Source data Fig. 1 [file 44319_2026_807_MOESM2_ESM.zip › Figure 1/1G/C3-MAX_DLD1_CIP2A KO #7_untrated (8).tif]

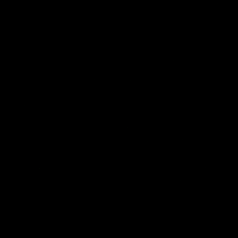

Supplement: Supplementary file 2 — Source data Fig. 1 [file 44319_2026_807_MOESM2_ESM.zip › Figure 1/1G/C1-MAX_DLD1_CIP2A KO #7_APH (12).tif]

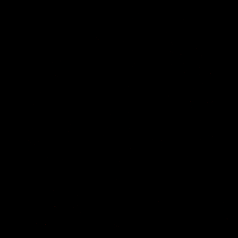

Supplement: Supplementary file 2 — Source data Fig. 1 [file 44319_2026_807_MOESM2_ESM.zip › Figure 1/1G/C3-MAX_DLD1_CIP2A KO #7_APH (12).tif]

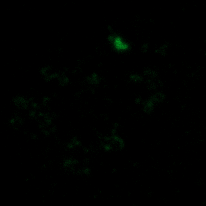

Supplement: Supplementary file 2 — Source data Fig. 1 [file 44319_2026_807_MOESM2_ESM.zip › Figure 1/1G/C1-MAX_DLD1_WT_untreated (10).tif]

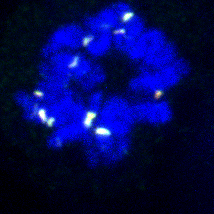

Supplement: Supplementary file 2 — Source data Fig. 1 [file 44319_2026_807_MOESM2_ESM.zip › Figure 1/1G/MAX_DLD1_WT_APH (4).tif (RGB).tif]

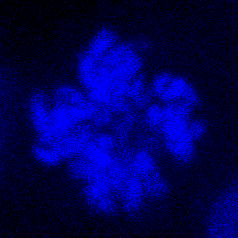

Supplement: Supplementary file 2 — Source data Fig. 1 [file 44319_2026_807_MOESM2_ESM.zip › Figure 1/1G/MAX_DLD1_CIP2A KO #7_APH (12).tif (RGB).tif]

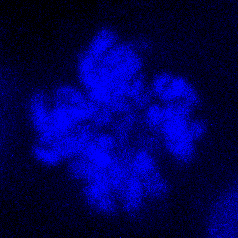

Supplement: Supplementary file 2 — Source data Fig. 1 [file 44319_2026_807_MOESM2_ESM.zip › Figure 1/1G/C2-MAX_DLD1_CIP2A KO #7_APH (12).tif]

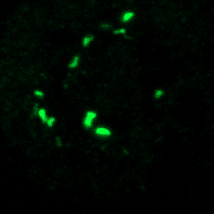

Supplement: Supplementary file 2 — Source data Fig. 1 [file 44319_2026_807_MOESM2_ESM.zip › Figure 1/1G/C1-MAX_DLD1_WT_APH (4).tif]

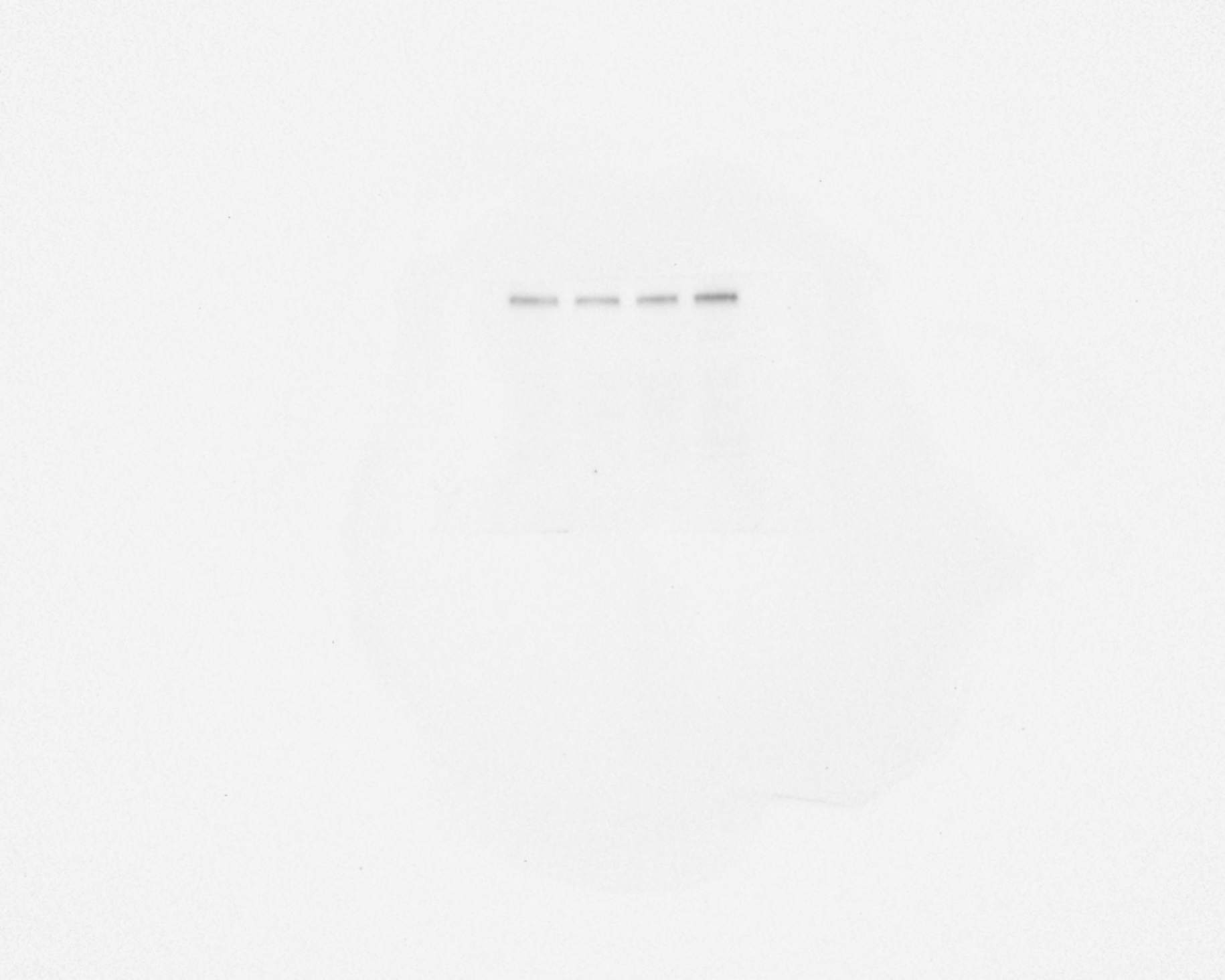

Supplement: Supplementary file 3 — Source data Fig. 2 [file 44319_2026_807_MOESM3_ESM.zip › Figure 2/2G/MUS81.tif]

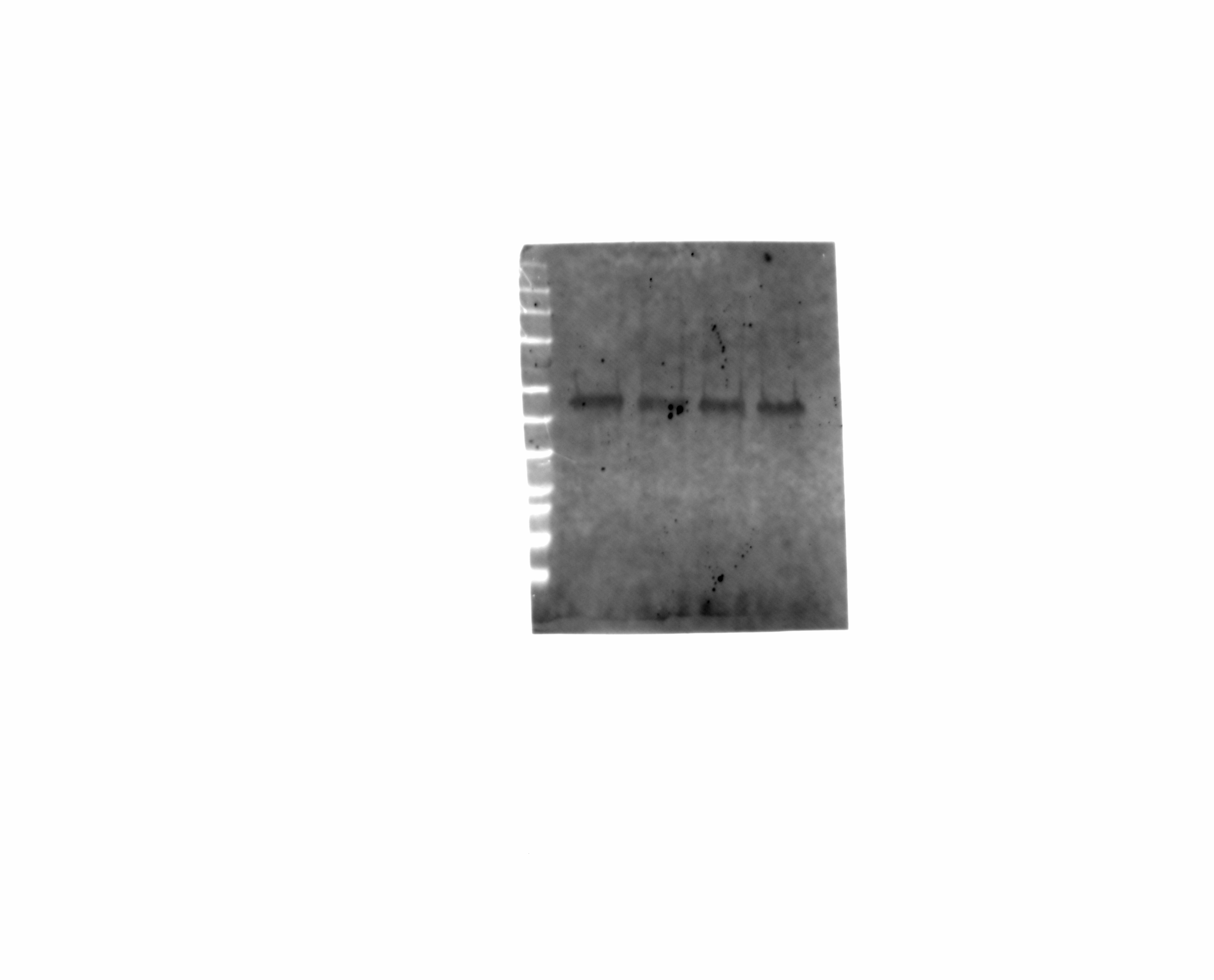

Supplement: Supplementary file 3 — Source data Fig. 2 [file 44319_2026_807_MOESM3_ESM.zip › Figure 2/2G/SLX4 TUBULIN.tif]

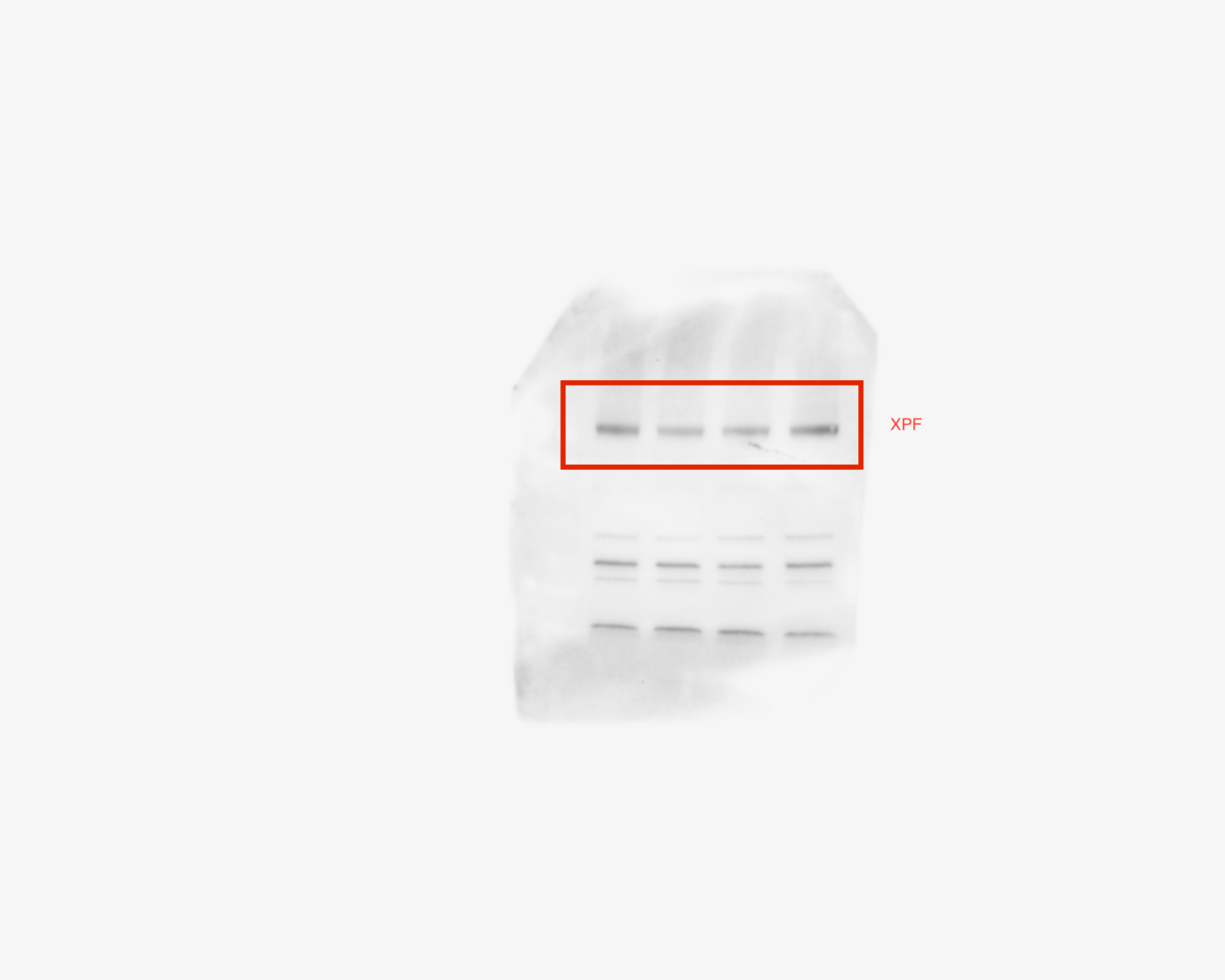

Supplement: Supplementary file 3 — Source data Fig. 2 [file 44319_2026_807_MOESM3_ESM.zip › Figure 2/2G/XPF.tif]

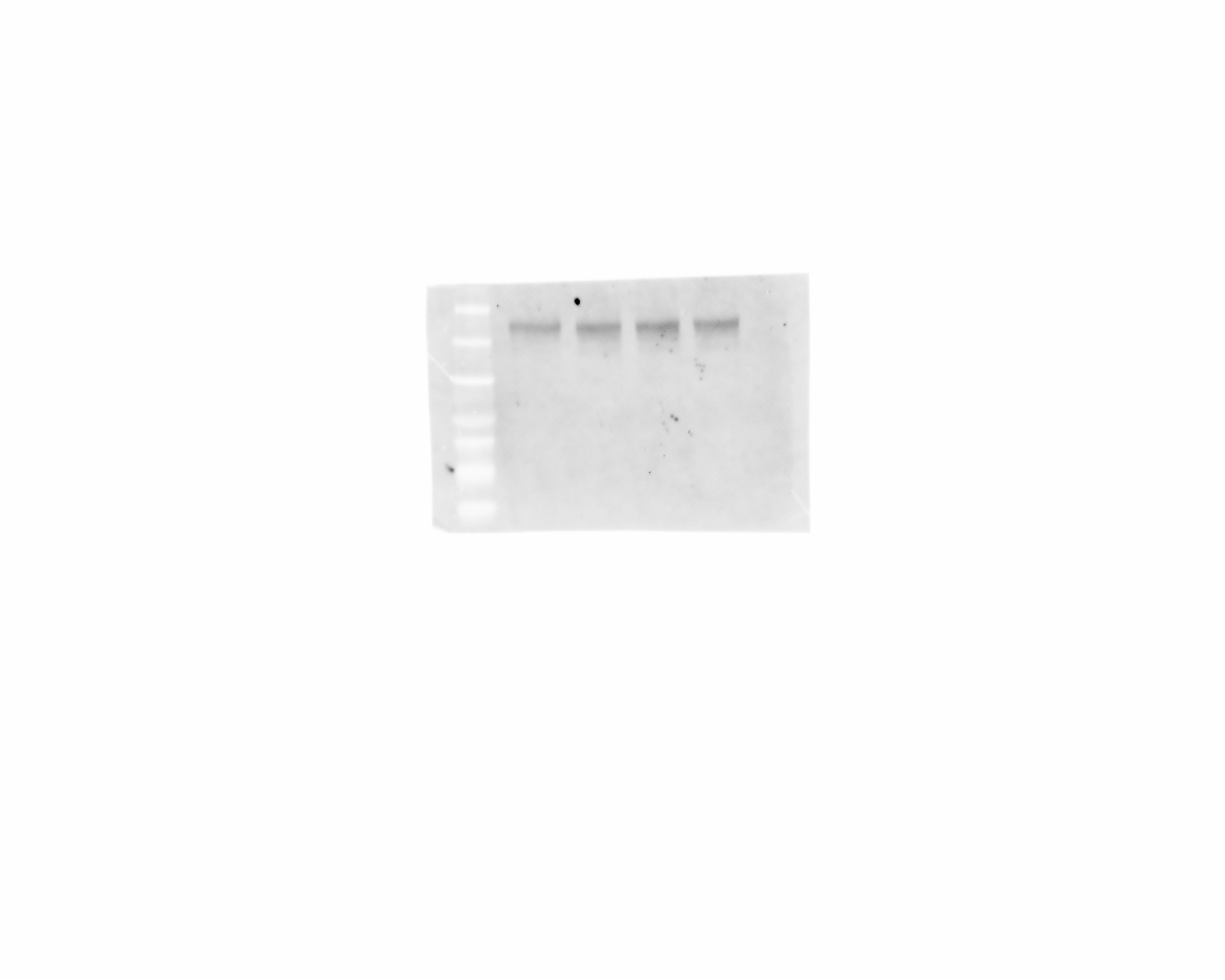

Supplement: Supplementary file 3 — Source data Fig. 2 [file 44319_2026_807_MOESM3_ESM.zip › Figure 2/2G/MUS81 TUBULIN.tif]

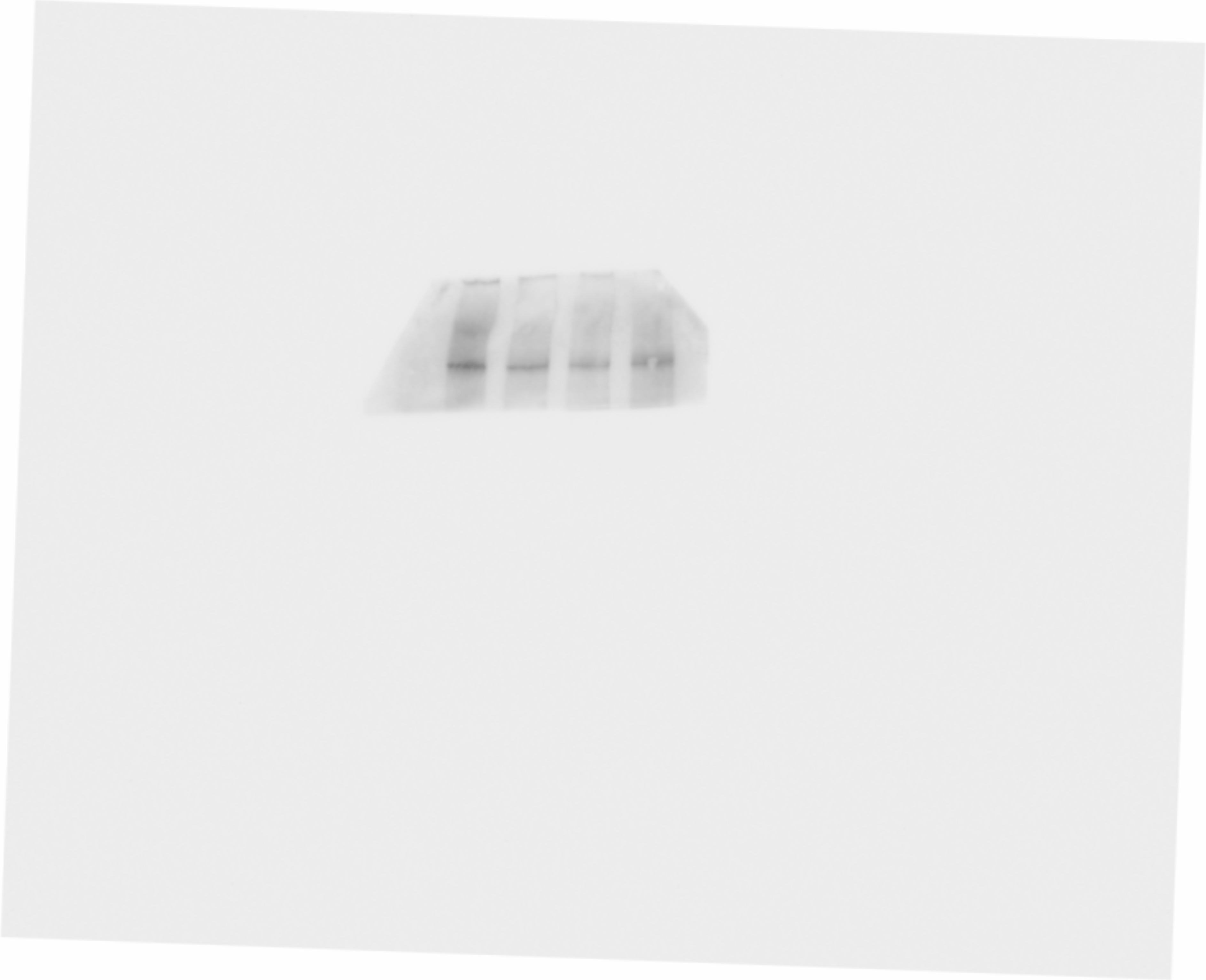

Supplement: Supplementary file 3 — Source data Fig. 2 [file 44319_2026_807_MOESM3_ESM.zip › Figure 2/2G/SLX4.tif]

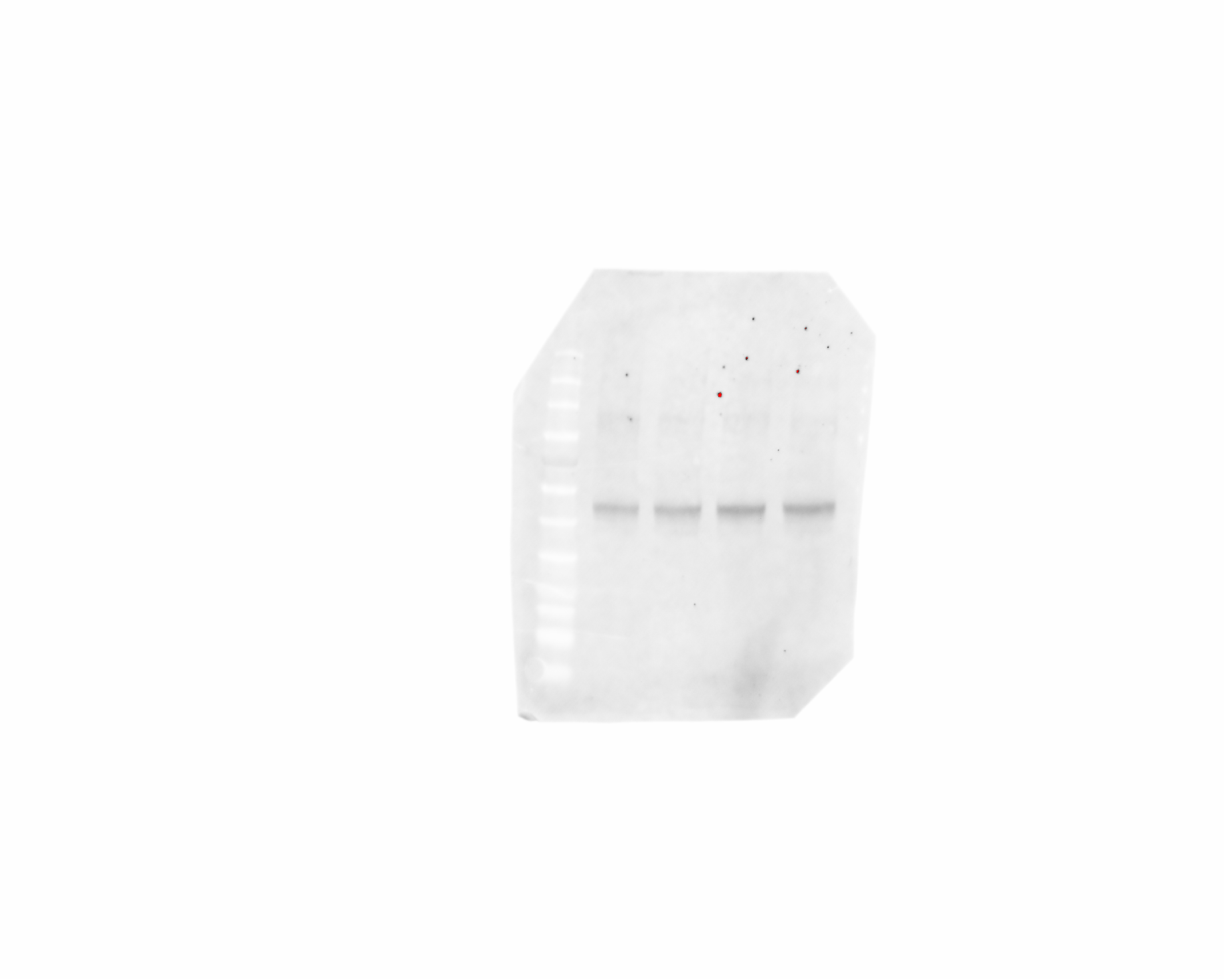

Supplement: Supplementary file 3 — Source data Fig. 2 [file 44319_2026_807_MOESM3_ESM.zip › Figure 2/2G/XPF TUBULIN.tif]

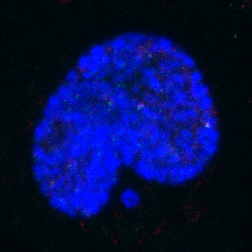

Supplement: Supplementary file 3 — Source data Fig. 2 [file 44319_2026_807_MOESM3_ESM.zip › Figure 2/2B/merge-U2OS_siCIP2A_APH_0522-1536.tif (RGB).tif]

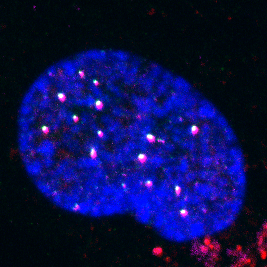

Supplement: Supplementary file 3 — Source data Fig. 2 [file 44319_2026_807_MOESM3_ESM.zip › Figure 2/2B/merge-U2OS_siCTRL_APH_0617-0646.tif (RGB).tif]

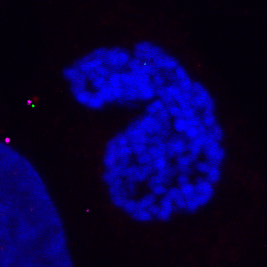

Supplement: Supplementary file 3 — Source data Fig. 2 [file 44319_2026_807_MOESM3_ESM.zip › Figure 2/2B/merge-U2OS_siCIP2A_UT_0258-1050.tif (RGB).tif]

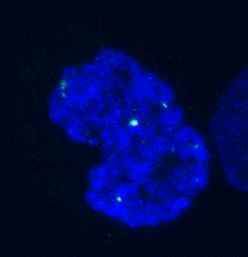

Supplement: Supplementary file 3 — Source data Fig. 2 [file 44319_2026_807_MOESM3_ESM.zip › Figure 2/2B/merge-U2OS_siCTRL_UT_0129-0645.tif (RGB).tif]

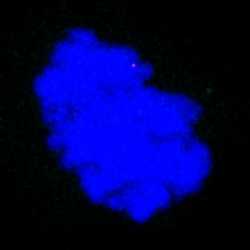

Supplement: Supplementary file 4 — Source data Fig. 3 [file 44319_2026_807_MOESM4_ESM.zip › Figure 3/3A/U2OS_siCIP2A_UT (RGB).tif]

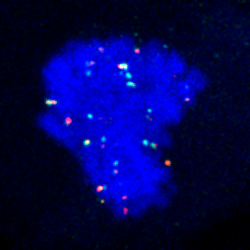

Supplement: Supplementary file 4 — Source data Fig. 3 [file 44319_2026_807_MOESM4_ESM.zip › Figure 3/3A/U2OS_siCTRL_Aph (RGB).tif]

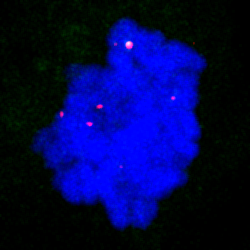

Supplement: Supplementary file 4 — Source data Fig. 3 [file 44319_2026_807_MOESM4_ESM.zip › Figure 3/3A/U2OS_siCTRL_UT (RGB).tif]

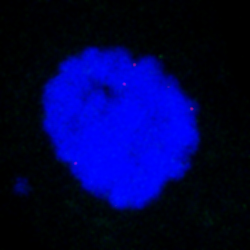

Supplement: Supplementary file 4 — Source data Fig. 3 [file 44319_2026_807_MOESM4_ESM.zip › Figure 3/3A/U2OS_siCIP2A_Aph (RGB).tif]

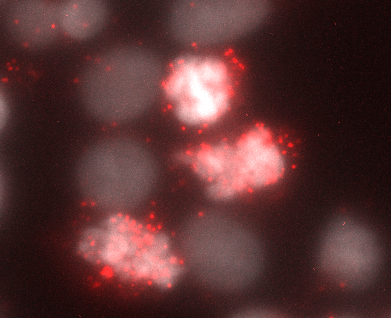

Supplement: Supplementary file 5 — Source data Fig. 4 [file 44319_2026_807_MOESM5_ESM.zip › Figure 4/4B/WT_unt.lif - Series006 CROP.tif (RGB).tif]

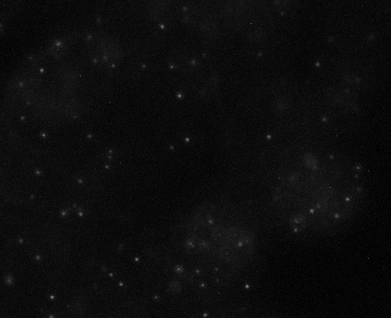

Supplement: Supplementary file 5 — Source data Fig. 4 [file 44319_2026_807_MOESM5_ESM.zip › Figure 4/4B/C2-WT_S1.lif - Series001 CROP.tif]

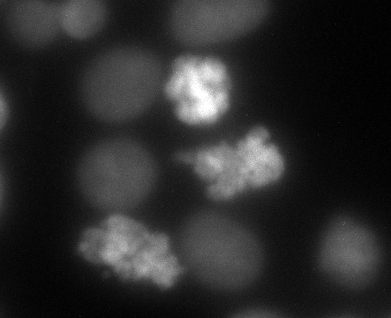

Supplement: Supplementary file 5 — Source data Fig. 4 [file 44319_2026_807_MOESM5_ESM.zip › Figure 4/4B/WT_unt.lif - Series006 CROP.tif]

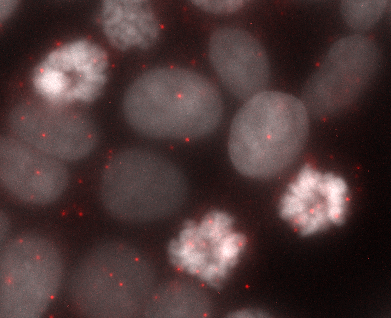

Supplement: Supplementary file 5 — Source data Fig. 4 [file 44319_2026_807_MOESM5_ESM.zip › Figure 4/4B/WT_S1.lif - Series001 CROP.tif (RGB).tif]

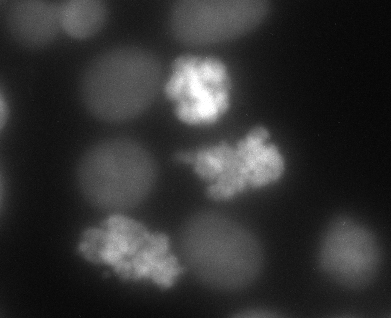

Supplement: Supplementary file 5 — Source data Fig. 4 [file 44319_2026_807_MOESM5_ESM.zip › Figure 4/4B/C1-WT_unt.lif - Series006 CROP.tif]

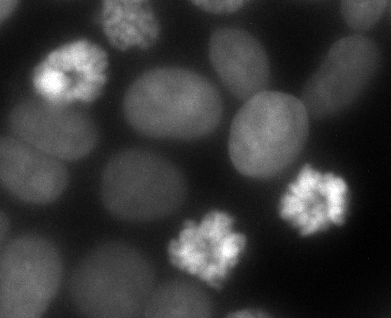

Supplement: Supplementary file 5 — Source data Fig. 4 [file 44319_2026_807_MOESM5_ESM.zip › Figure 4/4B/WT_S1.lif - Series001 CROP.tif]

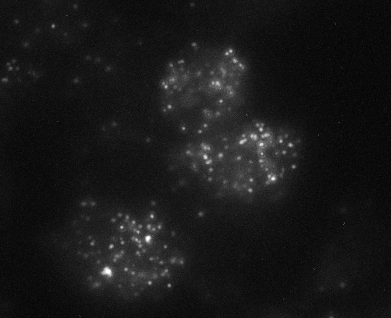

Supplement: Supplementary file 5 — Source data Fig. 4 [file 44319_2026_807_MOESM5_ESM.zip › Figure 4/4B/C2-WT_unt.lif - Series006 CROP.tif]

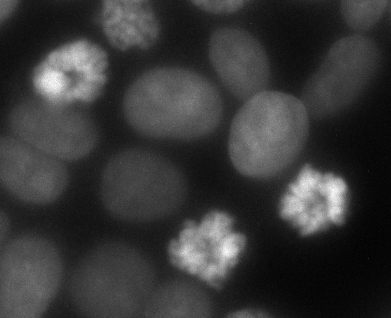

Supplement: Supplementary file 5 — Source data Fig. 4 [file 44319_2026_807_MOESM5_ESM.zip › Figure 4/4B/C1-WT_S1.lif - Series001 CROP.tif]

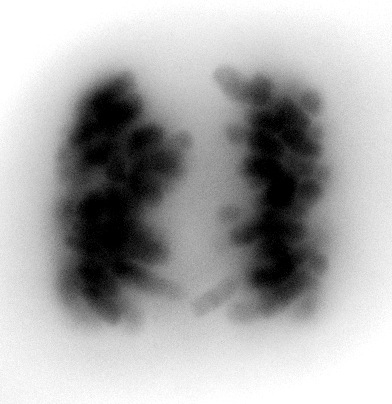

Supplement: Supplementary file 6 — Source data Fig. 5 [file 44319_2026_807_MOESM6_ESM.zip › Figure 5/5A/IF81H-siCTRL_UT-02_processed_s07c1-3.tif (blue).tif]

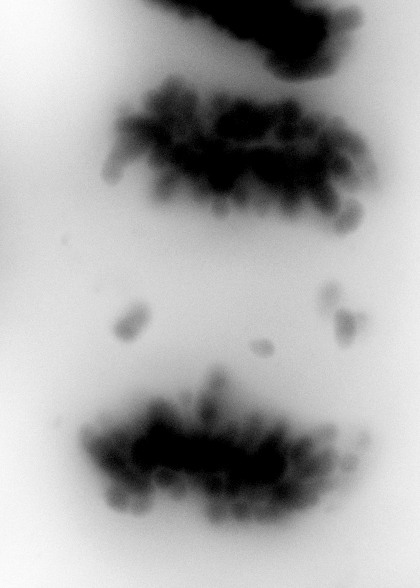

Supplement: Supplementary file 6 — Source data Fig. 5 [file 44319_2026_807_MOESM6_ESM.zip › Figure 5/5A/IF81H-siCIP2A_APH-01_processed_s04c1-3.tif (blue).tif]

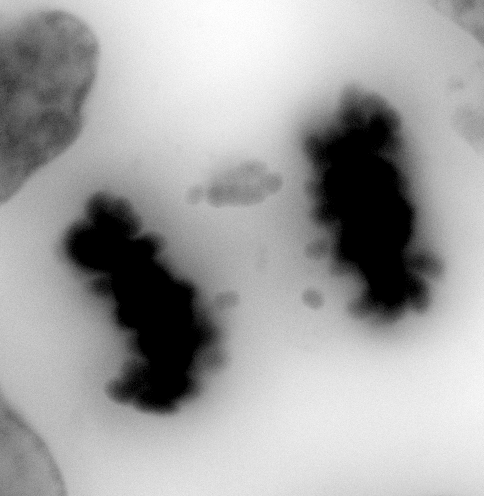

Supplement: Supplementary file 6 — Source data Fig. 5 [file 44319_2026_807_MOESM6_ESM.zip › Figure 5/5A/IF81H-siCIP2A_APH-01_processed_s04c1-4.tif (blue).tif]

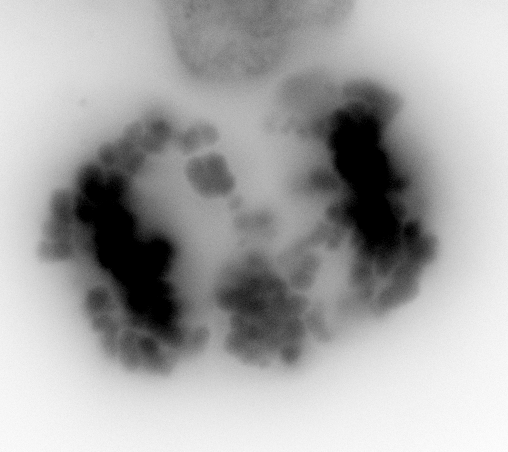

Supplement: Supplementary file 6 — Source data Fig. 5 [file 44319_2026_807_MOESM6_ESM.zip › Figure 5/5A/IF81H-siCIP2A_APH-01_processed_s21c1-3.tif (blue).tif]
