# Supplementary material for: CIP2A recruits SLX4-MUS81-XPF in mitosis and protects against replication stress
Source: EMBO Rep. 2026 May 26;27(13):3585–603. doi: 10.1038/s44319-026-00807-3 (PMC13354792; doi:10.1038/s44319-026-00807-3)
Supplement: Supplementary file 7 — Expanded View Figures [file 44319_2026_807_MOESM7_ESM.pdf]

Expanded View Figures

Figure EV1. The CIP2A-TOPBP1 complex responds to replication stress across multiple cell lines.

(**A**) Dot plot showing number of TOPBP1 foci per mitotic cell in DLD1 WT and DLD1 BRCA2 KO. Replicate medians (black dots) and the pooled median (black bar) are indicated. $n = 3$ biological replicates. Statistical analysis: Mann–Whitney test. (**B**) Western blot showing total protein levels of CIP2A in DLD1 BRCA2 KO cells following transfection with control siRNA (siCTRL) or siCIP2A. $n = 2$ biological replicates; a representative blot is shown. (**C**) Dot plot showing number of TOPBP1 foci per mitotic cell in CAPAN-1 and CAPAN-1 complemented with wild-type BRCA2 (+BRCA2). Replicate medians (black dots) and the pooled median (black bar) are indicated. $n = 3$ biological replicates. Statistical analysis: Mann–Whitney test. (**D**) Dot plot showing CIP2A foci per mitotic cell in RPE-1 treated with aphidicolin (Aph). Cells are treated or not with 0.4 μM aphidicolin for 24 h. In the last 6 h of treatment, CDK1 inhibitor RO-3306 is added at 7 μM. Cells are then washed with PBS, released into fresh media and collected after 25 min. Replicate medians (black dots) and the pooled median (black bar) are indicated. $n = 3$ biological replicates. Statistical analysis: Mann–Whitney test. (**E**) Dot plot showing CIP2A foci per mitotic cell in RPE-1 p53 KO treated with aphidicolin (Aph). Cells are treated or not with 0.4 μM aphidicolin for 24 h. In the last 6 h of treatment, CDK1 inhibitor RO-3306 is added at 7 μM. Cells are then washed with PBS, released into fresh media and collected after 25 min. Replicate medians (black dots) and the pooled median (black bar) are indicated. $n = 3$ biological replicates. Statistical analysis: Mann–Whitney test. For all the panels, data are presented with the following significance thresholds: ns (not significant), $P < 0.05$ (*), $P < 0.01$ (**), $P < 0.001$ (***), and $P < 0.0001$ (****).

© The Author(s)

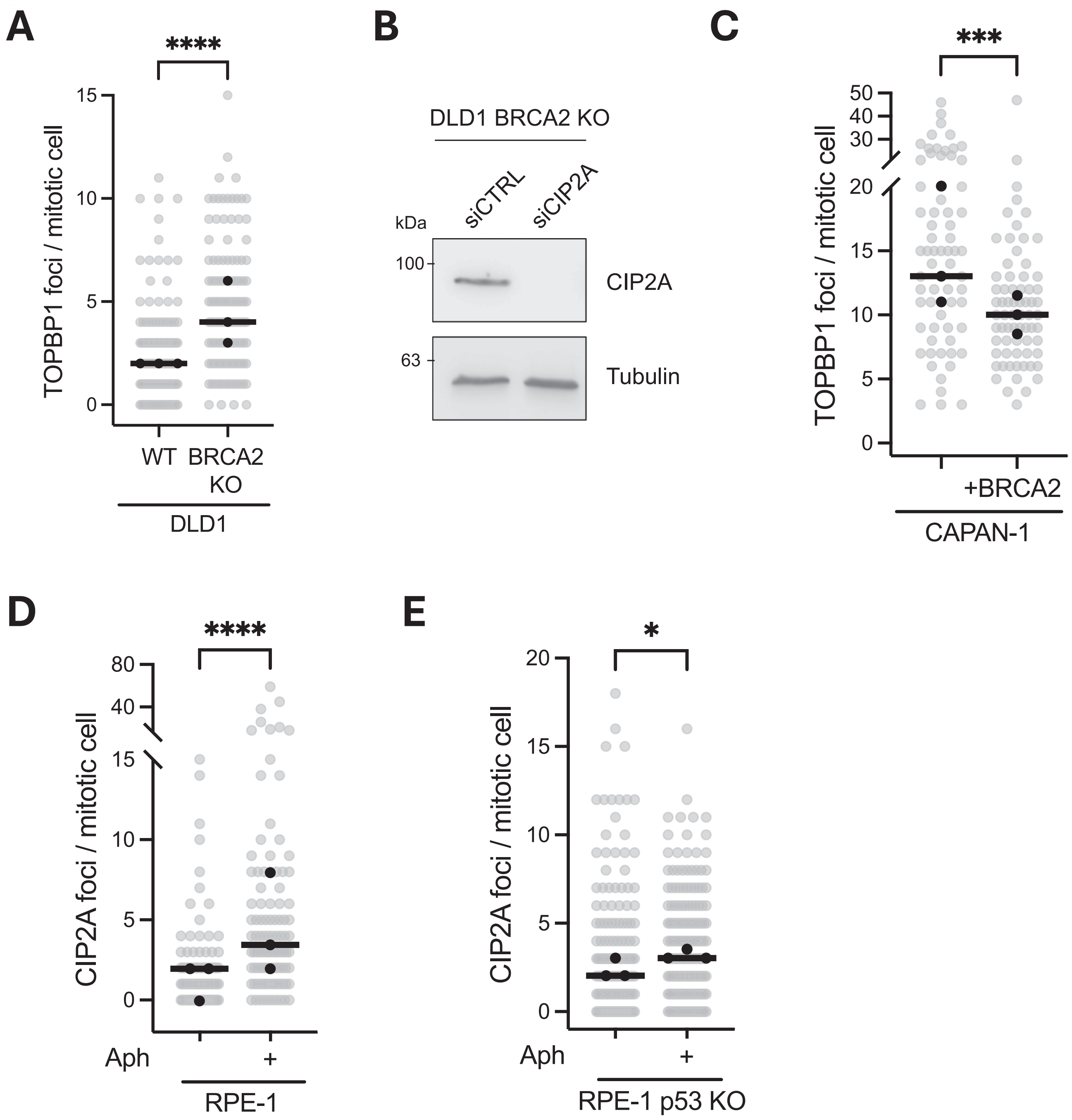

A
****
TOPBP1 foci / mitotic cell
15
10
5
0
WT
BRCA2 KO
DLD1
B
DLD1 BRCA2 KO
kDa
siCTRL
siCIP2A
100
CIP2A
63
Tubulin
C
***
TOPBP1 foci / mitotic cell
50
40
30
20
15
10
5
0
+BRCA2
CAPAN-1
D
****
CIP2A foci / mitotic cell
80
40
15
10
5
0
Aph
+
RPE-1
E
*
CIP2A foci / mitotic cell
20
15
10
5
0
Aph
+
RPE-1 p53 KO

 © The Author(s)

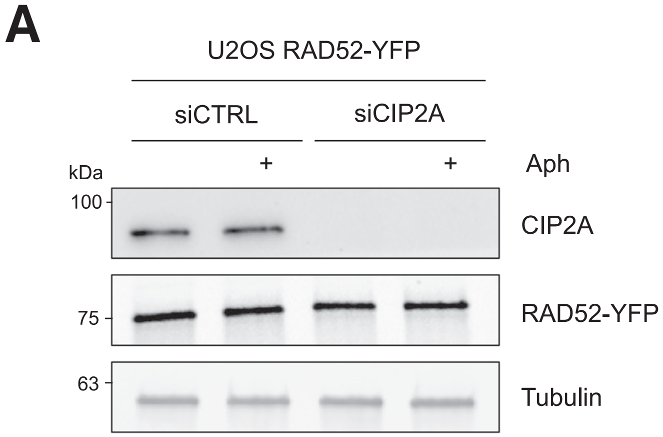

Figure EV2. CIP2A silencing in U2OS RAD52-YFP cells.

(**A**) Western blot showing total protein levels of CIP2A and RAD52-YFP in U2OS RAD52-YFP cells treated or not with aphidicolin following transfection with control siRNA (siCTRL) or siCIP2A.

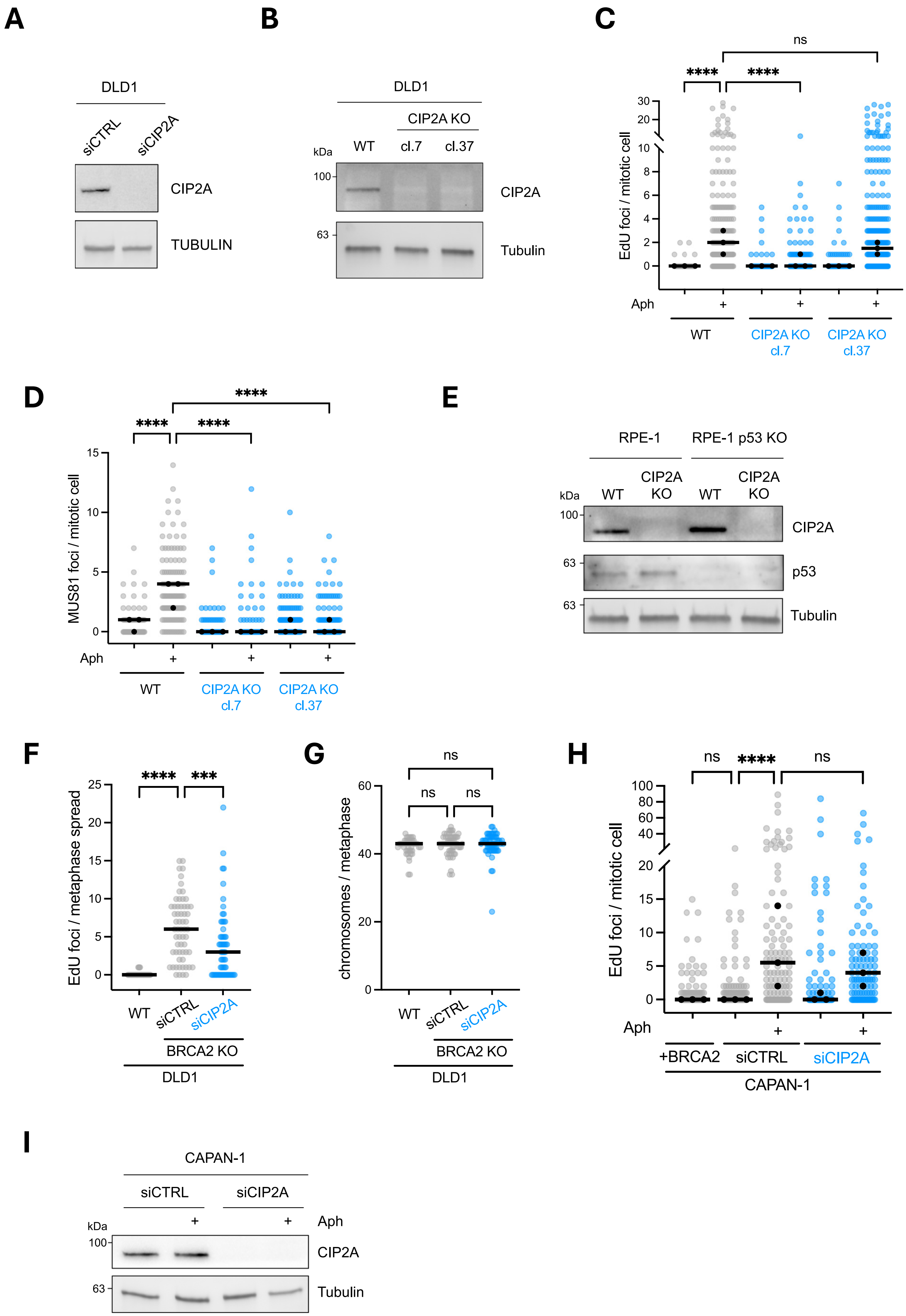
A
DLD1
siCTRL
siCIP2A
CIP2A
TUBULIN
B
DLD1
CIP2A KO
kDa
WT
cl.7
cl.37
100
CIP2A
63
Tubulin
C
ns
****
****
EdU foci / mitotic cell
Aph
WT
CIP2A KO cl.7
CIP2A KO cl.37
D
****
****
****
MUS81 foci / mitotic cell
Aph
WT
CIP2A KO cl.7
CIP2A KO cl.37
E
RPE-1
RPE-1 p53 KO
kDa
WT
CIP2A KO
WT
CIP2A KO
100
CIP2A
63
p53
63
Tubulin
F
****
***
EdU foci / metaphase spread
WT
siCTRL
siCIP2A
BRCA2 KO
DLD1
G
ns
ns
ns
chromosomes / metaphase
WT
siCTRL
siCIP2A
BRCA2 KO
DLD1
H
ns
****
ns
EdU foci / mitotic cell
Aph
+BRCA2
siCTRL
siCIP2A
CAPAN-1
I
CAPAN-1
siCTRL
siCIP2A
Aph
kDa
100
CIP2A
63
Tubulin

 © The Author(s)

Figure EV3. Additional analyses of CIP2A dependency for MiDAS across cell lines.

(**A**) Western blot showing total protein levels of CIP2A in DLD1 cells following transfection with control siRNA (siCTRL) or siCIP2A. (**B**) Western blot showing total protein levels of CIP2A in DLD1 WT, CIP2A KO cl.7 and c. 37. (**C**) Dot plot showing number of EdU foci per mitotic cell in DLD1 WT, CIP2A KO cl.7 and c. 37 treated as in Fig. 3A but collected after 25 min. Replicate medians (black dots) and the pooled median (black bar) are indicated. $n = 3$ biological replicates. Statistical analysis: Kruskal–Wallis test. (**D**) Dot plot showing number of MUS81 foci per mitotic cell in DLD1 WT, CIP2A KO cl.7 and c. 37 treated as in Fig. 3A but collected after 25 min. Replicate medians (black dots) and the pooled median (black bar) are indicated. $n = 3$ biological replicates. Statistical analysis: Kruskal–Wallis test. (**E**) Western blot showing total protein levels of CIP2A and p53 in RPE-1, RPE-1 CIP2A KO, RPE-1 p53 KO, RPE-1 p53 KO CIP2A KO. (**F**) Dot plot showing number of EdU foci per metaphase spread in DLD1 WT and BRCA2 KO, following transfection with control siRNA (siCTRL) or siCIP2A. Cells were synchronized with RO-3306 for 4 h, then released in presence of Colcemid and EdU for 1 h and collected. Pooled median (black bar) indicated. $n = 2$ biological replicates. Statistical analysis: Kruskal–Wallis test. (**G**) Dot plot showing number of chromosomes per metaphase spread in DLD1 WT and BRCA2 KO, following transfection with control siRNA (siCTRL) or siCIP2A. Pooled median (black bar) indicated. $n = 2$ biological replicates. Statistical analysis: Kruskal–Wallis test. (**H**) Dot plot showing number of EdU foci per mitotic cell in CAPAN-1 + BRCA2 and CAPAN-1, following transfection with control siRNA (siCTRL) or siCIP2A, treated as in Fig. 3A. Replicate medians (black dots) and the pooled median (black bar) are indicated. $n = 3$ biological replicates. Statistical analysis: Kruskal–Wallis test. (**I**) Western blot showing total protein levels of CIP2A in CAPAN-1 cells treated or not with aphidicolin following transfection with control siRNA (siCTRL) or siCIP2A. $n = 2$ biological replicates; a representative blot is shown. For all the panels, data are presented with the following significance thresholds: ns (not significant), $P < 0.05$ (*), $P < 0.01$ (**), $P < 0.001$ (***), and $P < 0.0001$ (****).

© The Author(s)
