# Supplementary material for: CIP2A recruits SLX4-MUS81-XPF in mitosis and protects against replication stress
Source: EMBO Rep. 2026 May 26;27(13):3585–603. doi: 10.1038/s44319-026-00807-3 (PMC13354792; doi:10.1038/s44319-026-00807-3)
Supplement: Supplementary file 1 — Peer Review File [file 44319_2026_807_MOESM1_ESM.pdf]

EMBO reports

CIP2A recruits SLX4-MUS81-XPF in Mitosis and Protects Against Replication Stress

Alice Meroni, Annica Pellizzari, Nathalie Varisco, Pascale Brasier-Lutz, Isabell Witzel, Alessandro Sartori, Manuel Stucki, Francesco Leone, Giada Greco, and Andrea Haenel

Corresponding author(s): Manuel Stucki (manuel.stucki@uzh.ch) , Alice Meroni (alice.meroni@uzh.ch)

Review Timeline:

Submission Date:	9th Dec 25
Editorial Decision:	17th Dec 25
Revision Received:	27th Mar 26
Editorial Decision:	27th Apr 26
Revision Received:	5th May 26
Accepted:	13th May 26

Editor: Esther Schnapp

Transaction Report:

(Note: With the exception of the correction of typographical or spelling errors that could be a source of ambiguity, letters and reports are not edited. Depending on transfer agreements, referee reports obtained elsewhere may or may not be included in this compilation. Referee reports are anonymous unless the Referee chooses to sign their reports.)

1st Editorial Decision 17th Dec 2025

Dear Prof. Stucki,

Thank you for the transfer of your peer-reviewed manuscript to EMBO reports, and for your revision plan.

I agree with your plan for how to revise your ms and would thus like to invite you to revise it with the understanding that the referee concerns must be fully addressed and their suggestions taken on board. Please address all referee concerns in a complete point-by-point response. Acceptance of the manuscript will depend on a positive outcome of a second round of review. It is EMBO reports policy to allow a single round of major revision only and acceptance or rejection of the manuscript will therefore depend on the completeness of your responses included in the next, final version of the manuscript.

We realize that it is difficult to revise to a specific deadline. In the interest of protecting the conceptual advance provided by the work, we recommend a revision within 2 months (19th Feb 2026). Would this work for you? If you can submit the revised study even earlier, this is better of course, given the very recent already published and related papers.

You can either publish the study as a short report or as a full article. For short reports, the revised manuscript should not exceed 29,000 characters (including spaces but excluding materials & methods and references) and 5 main plus 5 expanded view figures. The results and discussion sections must further be combined, which will help to shorten the manuscript text by eliminating some redundancy that is inevitable when discussing the same experiments twice. For a normal article there are no length limitations, but it should have more than 5 main figures and the results and discussion sections must be separate. In both cases, the entire materials and methods must be included in the main manuscript file.

IMPORTANT NOTE: we perform an initial quality control of all revised manuscripts before re-review. Your manuscript will FAIL this control and the handling will be DELAYED if the following APPLIES:
1) A data availability section providing access to data deposited in public databases is missing. If you have not deposited any data, please add a sentence to the data availability section that explains that.
2) Your manuscript contains statistics and error bars based on n=2. Please use scatter blots in these cases. No statistics should be calculated if n=2.

When submitting your revised manuscript, please review the submission guidelines (https://link.springer.com/journal/44319/submission-guidelines#cms-Revised-submissions) and carefully review the instructions that follow below. Failure to include requested items will delay the evaluation of your revision.

1) a .docx formatted version of the manuscript text (including legends for main figures, EV figures and tables). Please make sure that the changes are highlighted to be clearly visible.

2) individual production quality figure files as .eps, .tif, .jpg (one file per figure). See hhttps://media.springernature.com/original/springer-cms/rest/v1/content/27825798/data/v1 for more info on how to prepare your figures.

3) We replaced Supplementary Information with Expanded View (EV) Figures and Tables that are collapsible/expandable online. A maximum of 5 EV Figures can be typeset. EV Figures should be cited as 'Figure EV1, Figure EV2" etc... in the text and their respective legends should be included in the main text after the legends of regular figures.

- For the figures that you do NOT wish to display as Expanded View figures, they should be bundled together with their legends in a single PDF file called *Appendix*, which should start with a short Table of Content. Appendix figures should be referred to in the main text as: "Appendix Figure S1, Appendix Figure S2" etc. See 'Expanded View' section of the submission guidelines.

- Additional Tables/Datasets should be labeled and referred to as Table EV1, Dataset EV1, etc. Legends have to be provided in a separate tab in case of .xls files. Alternatively, the legend can be supplied as a separate text file (README) and zipped together with the Table/Dataset file.

4) a .docx formatted letter INCLUDING the reviewers' reports and your detailed point-by-point responses to their comments. As part of the EMBO Press transparent editorial process, the point-by-point response is part of the Review Process File (RPF), which will be published alongside your paper.

5) a complete author checklist, which you can download from our author guidelines. Please insert information in the checklist that is also reflected in the manuscript. The completed author checklist will also be part of the RPF.

6) Please note that all corresponding authors are required to supply an ORCID ID for their name upon submission of a revised manuscript (), which can be linked to your profile in the manuscript submission system.

7) Before submitting your revision, primary datasets produced in this study need to be deposited in an appropriate public

database. Please remember to provide a reviewer password if the datasets are not yet public. The accession numbers and database should be listed in a formal "Data Availability" section placed after Methods. Please note that the Data Availability Section is restricted to new primary data that are part of this study. * Note - All links should resolve to a page where the data can be accessed. *

If your study has not produced novel datasets, please mention this fact in the Data Availability Section.

8) At EMBO Press we ask authors to provide source data for the main manuscript figures. You will receive a separate email with instructions for providing source data with your revised manuscript, including how to upload and organize the files.

9) Our journal also encourages inclusion of *data citations in the reference list* to directly cite datasets that were re-used and obtained from public databases. Data citations in the article text are distinct from normal bibliographical citations and should directly link to the database records from which the data can be accessed. In the main text, data citations are formatted as follows: "Data ref: Smith et al, 2001" or "Data ref: NCBI Sequence Read Archive PRJNA342805, 2017". In the Reference list, data citations must be labeled with "[DATASET]". A data reference must provide the database name, accession number/identifiers and a resolvable link to the landing page from which the data can be accessed at the end of the reference.

10) Regarding data quantification, the following points must be specified in each figure legend:

- the name of the statistical test used to generate error bars and P values,

- the number (n) of independent experiments (please specify technical or biological replicates) underlying each data point,

- the nature of the bars and error bars (s.d., s.e.m.),

- If the data are obtained from n Program fragment delivered error ``Can't locate object method "less" via package "than" (perhaps you forgot to load "than"?) at //ejpvfs23/sites23b/embor_www/letters/embor_decision_rc_revise_and_rereview.txt line 54.' 2, use scatter blots showing the individual data points.

Discussion of statistical methodology can be reported in the materials and methods section, but figure legends should contain a basic description of n, P and the test applied.

- Please also include scale bars in all microscopy images.

11) The journal requires a statement specifying whether or not authors have competing interests (defined as all potential or actual interests that could be perceived to influence the presentation or interpretation of an article). In case of competing interests, this must be specified in your disclosure statement.

12) All Materials and Methods need to be described in the main text using our 'Structured Methods' format, which is required for all research articles. According to this format, the Methods section includes a separate Reagents and Tools Table file (listing key reagents, experimental models, software and relevant equipment and including their sources and relevant identifiers) and a Methods and Protocols section describing the methods using a step-by-step protocol format. The aim is to facilitate adoption of the methodologies across labs. More information on how to adhere to this format as well as a downloadable template (.docx) for the Reagents and Tools Table can be found in our author guidelines.

We would also welcome the submission of cover suggestions, or motifs to be used by our Graphics Illustrator in designing a cover.

As part of the EMBO publication's Transparent Editorial Process, EMBO reports publishes online a Review Process File (RPF) to accompany accepted manuscripts. This File will be published in conjunction with your paper and will include the referee reports, your point-by-point response and all pertinent correspondence relating to the manuscript.

You are able to opt out of this by letting the editorial office know (emboreports@embo.org). If you do opt out, the Review Process File link will point to the following statement: "No Review Process File is available with this article, as the authors have chosen not to make the review process public in this case."

I look forward to seeing a revised form of your manuscript when it is ready.

Best regards,
Esther

Esther Schnapp, PhD
Senior Editor
EMBO reports

Point-by-point Response to Reviewers

Note: Reviewer's comments are in *italic*, our responses are in normal text. All changes in the manscript text and Figure legends have been marked in red.

Reviewer #1

SUMMARY & SIGNIFICANCE

This is a timely and exciting study that provides us with some new molecular insights into mitotic DNA repair. It builds on previous studies that identified the CIP2A-TOPBP1 complex as a molecular tether that connects broken DNA ends that get transmitted from interphase into mitosis (PMID: 30898438, 35842428, 35842428). The results are also largely complementary with those of Martin et al. (BioRxiv preprint at https://doi.org/10.1101/2024.11.12.621593) and de Haan et al. (BioRxiv preprint at https://www.biorxiv.org/content/10.1101/2025.04.03.647079v1).

The authors report three main findings, as summarized below.

1) The CIP2A oncoprotein is involved in the cellular response to replication stress in mitosis.

2) CIP2A is required for the recruitment of SLX4, MUS81, and XPF into foci during mitosis. SLX4 is a well-established protein scaffold for multiple DNA repair factors, including three structure-selective endonucleases called SLX1, MUS81-EME1, and XPF-ERCC1 that together, form the SMX tri-nuclease that removes DNA repair intermediates and chromosome entanglements during mitosis. In some cell lines, the SMX complex is required for mitotic DNA synthesis at sites of under-replicated DNA, thus ensuring complete DNA replication prior to cell division.

3) The role(s) of CIP2A in MiDAS are cell line-dependent/context-dependent.

In general, this is a solid body of microscopy-based work that includes appropriate cell models and experimental controls. The manuscript is well-written, and the data is presented coherently. The main findings will have important implications for researchers

interested in mitotic DNA damage, genome stability, and cancer biology. After addressing the points below, I believe this manuscript will be suitable for publication.

MAJOR COMMENTS

1) Figure 1C: The CIP2A-TOPBP1 PLA experiments are lacking critical controls, namely cells lacking or depleted of CIP2A and TOPBP1. These controls are necessary to provide confidence for the results presented in Figure 1C. If these controls are too expensive or time-demanding for the manuscript, then I recommend removing the PLA data from Figure 1C.

We thank the reviewer for raising this important point. We agree that the initial PLA experiment lacked essential specificity controls. Therefore, we repeated the PLA experiment independently three times and included an siCIP2A condition as a negative control. Depletion of CIP2A resulted in a strong reduction of the PLA signal, demonstrating that the detected signal depends on CIP2A interaction with TOPBP1 and supporting the specificity of the assay. Using this revised and properly controlled setup, we also confirmed that the PLA signal is enhanced in BRCA2 knockout cells, consistent with our original observation. The updated data, including the new controls, have now replaced the previous panel in **Figure 1B**.

2) In Figure 2, the authors conclude that the loss of SLX4, XPF, and MUS81 foci in CIP2A depleted cells is synonymous with the loss of recruitment to DNA lesions. However, I can think of many other reasons that could explain the loss of foci. For example, do the authors know that the proteins are expressed to similar levels in cells with and without CIP2A (this should be tested by a simple western blot). Along the same vein, a biochemical fractionation and western blot of the soluble vs chromatin-bound fraction would complement and substantiate their microscopy-based assays in Figure 2. If the fractionation is not possible, then the text should be adjusted accordingly.

We thank the reviewer for this important suggestion. To address this point, we assessed the total protein levels of SLX4, XPF, and MUS81 by western blot in control and CIP2A-depleted cells. We did not observe any significant differences in the steady-state levels of these proteins upon CIP2A depletion. To further complement our immunofluorescence analysis, we also examined total protein levels following aphidicolin treatment. Again, we did not detect any changes in SLX4, XPF, or MUS81 abundance in the presence or absence of CIP2A. These results indicate that the reduction in foci observed by microscopy is not due to altered protein expression or

stability but is consistent with a defect in recruitment at sites of mitotic DNA damage. We agree that this experiments substantially strengthens the interpretation of Figure 2; therefore, we have included these new data in the main manuscript (**Figure 2G**).

3) The experimental set-up in Figure 2 probes whether CIP2A mediates the recruitment of SMX subunits - SLX4, XPF, MUS81 - but not the SMX complex per se, which would require the study of SLX4 point mutants that selectively ablate the interactions with XPF or MUS81 (but not CIP2A). As such, I suggest that they rephrase their wording appropriately.

We thank the reviewer for this important clarification. We agree that our original wording overstated the conclusion, as the experiments initially assessed individual SMX subunits rather than the fully assembled complex. To more directly address whether CIP2A mediates recruitment of the SMX complex per se, we performed triple immunofluorescence staining for SLX4, XPF, and MUS81, taking advantage of antibodies raised in different species. We then quantified foci in which all three proteins colocalize using an automated CellProfiler pipeline, thereby restricting our analysis to sites likely representing assembled SMX complexes rather than individual subunits. Consistent with our single-subunit analyses, the number of triple-colocalizing foci was significantly reduced upon CIP2A depletion, following the same trend observed for the individual components. These results support the conclusion that CIP2A promotes the recruitment of the SMX complex to mitotic DNA lesions. We have incorporated these new data into the main manuscript (**Figures 2B and 2F**) and have revised the text throughout to more precisely reflect that our conclusions are based on colocalization of SMX components.

4) Western blots must be provided to substantiate the experiments performed with siRNA (Figure 1G-J, Figure 2A-E and 2H, Figure 3A-D, Figure 5B-D). Similarly, the authors should provide western blots to confirm the BRCA2 and CIP2A statuses in their KO cell lines, as well as the complementation cell lines. In the absence of this information, it is difficult for someone to make an independent and meaningful interpretation of their data.

We have now included western blot analyses confirming CIP2A depletion in all siRNA experiments, as well as validation of CIP2A status in the respective knockout cell lines. These data demonstrate efficient knockdown in the siRNA-treated samples and complete loss of protein expression in the corresponding KO cells. Because these data provide a more direct assessment of protein levels, we have removed the previously shown immunofluorescence-based validation experiments to avoid redundancy. All validation western blots are now included in the manuscript (**Figures 1H, EV1B, EV2A, EV3A, EV3B, EV3E**).

5) Most of the data presented in this manuscript is derived from n = 2 biological replicates. All of the experiments reported in the study should be repeated for n = 3 biological replicates.

All experiments included in the manuscript have now been performed with three independent biological replicates (n = 3). We have also included the median of each biological replicate directly on the plots as a “SuperPlot” representation. The figure legends and the Methods have been revised accordingly.

6) Since the authors report the median of their data, they should also report the interquartile range or confidence interval to display the uncertainty.

We thank the reviewer for this thoughtful suggestion. In our plots, all single-cell measurements are displayed, allowing direct visualization of the full distribution of the data. The spread of the population can therefore be assessed visually without the need for additional summary statistics. In addition, we now report the median of each independent biological replicate using a SuperPlot format, which makes inter-replicate variability explicit. Because both the full distribution and replicate-level medians are shown, we believe that adding interquartile ranges or confidence intervals would be redundant and could reduce figure clarity without providing additional informative value. Nevertheless, the statistical analysis performed is described in the figure legends and Methods section.

MINOR COMMENTS

1) The references can be improved by acknowledging some of the foundational papers on SLX4 and the SMX tri-nuclease.

1.a) Page 3: Neither Minocherhomji et al. 2015 nor Pedersen et al. 2015 were the first to describe SLX4 as a scaffold for structure-selective endonucleases. The founding papers were published in 2009 (Svendsen et al. 2009, Munoz et al. 2009, Fekairi et al. 2009, Andersen et al. 2009) with important mechanistic studies on nuclease activation reported in 2013 (Wyatt et al. 2013, Castor et al. 2013) and 2017 (Wyatt et al. 2017).

1.b) Page 6: The authors should cite Wyatt et al. 2013, alongside Castor et al. 2013 and Garner et al. 2013 since these 3 articles were published at similar times. They may also want to acknowledge previous work from the Hickson and Rosselli labs showing that XPF-ERCC1 and MUS81-EME1 are recruited to fragile sites in mitosis.

We have incorporated the recommended references.

2) To improve broad readability, the authors should remove the following abbreviations: Aph and WT.

We have removed the abbreviations “Aph” and “WT” throughout the manuscript text and replaced them with the full terms to improve clarity and readability.

3) In several figures, the authors show that a given treatment causes a very small change in the number of foci observed per mitotic cell. Although the values may be statistically different, it is important that they discuss the biological significance of these small effects - for example, I am not convinced that a difference of 2-3 foci per cell is sufficient to induce a robust cellular response.

We thank the reviewer for this important comment. We agree that small but statistically significant differences do not necessarily translate into biologically meaningful effects. We have therefore revised the text to more carefully discuss the magnitude of the observed changes and to avoid overstating their biological significance where the effect size is modest.

4) The methods could be expanded to ensure reproducibility, particularly with respect to the drug treatments (e.g., timing, washes, etc.).

The Methods section has been expanded, and more details are now provided to ensure the reproducibility of our experimental scheme.

Reviewer #1 (Significance (Required)):

This is a timely and exciting study that provides us with some new molecular insights into mitotic DNA repair. It builds on previous studies that identified the CIP2A-TOPBP1 complex as a molecular tether that connects broken DNA ends that get transmitted from interphase into mitosis (PMID: 30898438, 35842428, 35842428). The results are also largely complementary with those of Martin et al. (BioRxiv preprint at https://doi.org/10.1101/2024.11.12.621593) and de Haan et al. (BioRxiv preprint at https://www.biorxiv.org/content/10.1101/2025.04.03.647079v1).

Reviewer #2

Summary:

In the manuscript entitled "CIP2A Mediates the Recruitment of the SLX4-MUS81-XPF Tri-Nuclease Complex in Mitosis and Protects Against Replication Stress" by Meroni et al the authors have characterized localization of the CIP2A-TopBP1 complex as well as some aspects of its function in U2OS and DLD1 cell lines exposed to different types of stress. They find that replication stress due to BRCA2 KO, APH or ATRi results in increased focus formation of the CIP2A-TopBP1 complex in mitotic cells.

Moreover, the authors find significant decrease in EdU incorporartion in mitotic cells when disrupting CIP2A in (i) U2OS exposed to ATRi or Aph; (ii) in DLD1 BRCA2 KO; (iii) in one clone of DLD1 with Cip2A KO, and a non significant decrease the other DLD1 with Cip2A KO that they tested. Thus, under most of the tested conditions CIP2A is facilitating MiDAS. However, the authors find that expression of a previously characterised fragment of TopBP1 called B6L, which disrupts CIP2A-TopBP1 interaction, does not inhibit MiDAS in DLD1 cells.

Major comments:

It is convincing but not surprising that CIP2A-TopBP1 form more foci in mitotic cells after replication stress.

The authors statement in the abstract: "We demonstrate that in the absence of CIP2A, cells fail to recruit the SLX4-MUS81-XPF (SMX) tri-nuclease complex to sites of under-replicated DNA in mitosis, resulting in a

high incidence of lagging chromosomes during anaphase and subsequent micronuclei formation" is not supported by experiments. The authors indeed show that absence of CIP2A leads to lagging chromosomes during anaphase and subsequent micronuclei formation (which has previously been shown) but they have not shown that it is the failure to recruit the SMX complex that results in the phenotypes they mention. The authors should rephrase or remove this claim.

We thank the reviewer for this important comment. We agree that our previous wording implied a causal relationship that was not directly demonstrated by the experiments presented. We have therefore revised the abstract and removed this claim to avoid overinterpretation.

There is a discrepancy between the B6L-mediated disruption of TopBP1-CIP2A interaction having no effect on MiDAS in DLD1 cells (fig. 4F) whereas knockout of CIP2A in DLD1 cells seem to have an effect (fig 3E). The most obvious explanation for this observation is that the B6L peptide does not fully abolish TopBP1-CIP2A interaction and can still allow for some SLX4-MUS81 recruitment that is not visible as foci but still sufficient to induce MiDAS.

To understand whether MiDAS in DLD1 expressing B6L is dependent on the fraction of TopBP1 that can still form foci (according to Fig 4D) the authors must co-stain for TopBP1 together with EdU detection to address whether they observe any colocalization of TopBP1 with MiDAS.

From minor comments: In figure 4D - is the increase with APH and S1 significant compared to S1 alone?

We thank the reviewer for raising this point. We would like to clarify that the clonal variability in the DLD1 CIP2A KO cells was already present in the submitted manuscript. Specifically, we showed that MiDAS is completely abolished in CIP2A KO clone 7, whereas in clone 37 it is not significantly different from wild-type cells, indicating a clone-specific effect rather than a uniform consequence of CIP2A loss. We apologize if this distinction was not sufficiently clear in the initial version.

To further exclude clonal artifacts and strengthen the conclusion, we additionally repeated the experiment using siRNA-mediated depletion of CIP2A in DLD1 cells. Under these conditions, MiDAS was only modestly reduced. In contrast, MUS81 recruitment was consistently abolished in both KO clones and in siCIP2A-treated cells. These data indicate that CIP2A is crucial for MUS81 recruitment after replication stress, whereas MiDAS can occur, at least in part, independently of efficient MUS81 recruitment. To improve clarity, the data from the CIP2A KO clones have been moved to the Expanded View Figures (**Figure EV3B, C, D**), while the siCIP2A data are now presented in the main Figure (**Figure 3C, D**). The corresponding text has been revised accordingly.

Regarding the B6L-mediated disruption of the TOPBP1–CIP2A interaction, our data show that this interaction is effectively abolished after 24 hours of Shield-1 (S1) treatment (**Figure 4B, C**), as demonstrated by loss of the PLA signal and near-complete absence of CIP2A foci. However, as noticed by the reviewer, a small but statistically none-significant number of TOPBP1 foci remain detectable after S1 treatment. As suggested, we performed co-staining of EdU and TOPBP1 in three independent biological replicates, followed by Squassh colocalization analysis. Our data revealed no detectable colocalization between residual TOPBP1 foci and EdU-positive MiDAS sites (**Rev Figure1**), indicating that the remaining MiDAS events are not associated with TOPBP1 foci after disruption of the TOPBP1–CIP2A interaction.

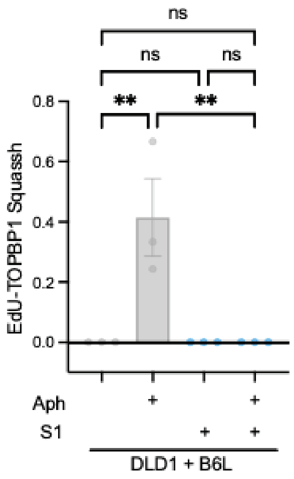

Rev Figure 1

Many of the experiments are only performed with two independent replicates. The authors must perform 3 independent replicates. Also, it is not clear how many cells were analysed for each replicate. This should be clearly stated and the mean of each replicate should always be shown. Statistical analyses should be carried out using the means of the replicates.

All experiments included in the manuscript have now been performed with three independent biological replicates (n = 3).

Importantly, single-cell measurement data were formally assessed for normality using a normality test, and they did not meet the assumptions of a Gaussian distribution. Therefore, we applied non-parametric statistical testing. Consistent with this approach, we report medians rather than means, as the median provides a more appropriate and robust measure of central tendency for non-normally distributed data and is less influenced by skewness or outliers. We have now included the median of each biological replicate directly on the plots as a “SuperPlot” representation and the figure legends have been revised accordingly.

The number of cells analyzed per replicate is specified in the Methods (Immunofluorescence and Image analysis section). In general, between 30 and 80 cells were quantified per condition per biological replicate.

The authors must provide data showing the efficiency of CIP2A knockdown and CIP2A expression in the complementation assay (Fig. 2G)

We have now included western blot analyses confirming CIP2A depletion in all siRNA experiments, as well as validation of CIP2A status in the respective knockout cell lines. These data demonstrate efficient knockdown in the siRNA-treated samples and

complete loss of protein expression in the corresponding KO cells. Because these data provide a more direct assessment of protein levels, we have removed the previously shown immunofluorescence-based validation experiments to avoid redundancy. All validation western blots are now included in the Extended View Figures (**Figures 1H, EV1B, EV2A, EV3A, EV3B, EV3E**).

Minor comments:

The authors should change "U-2 OS" in the figures to "U2OS" for consistency.

All instances of “U-2 OS” in the figures have been updated to “U2OS” for consistency throughout the manuscript.

Figure 3 B and C. It is worrying that there is a huge difference in the EdU foci/mitotic cell in untreated condition from panel B to pabel C.

We thank the reviewer for pointing out these discrepancies. Upon careful review, we believe these differences likely arose because the original experiments were performed by multiple researchers, which may have introduced subtle inconsistencies in experimental handling, timing, or imaging settings. To eliminate these sources of variability, we repeated the experiment from scratch, ensuring that it was performed by a single researcher under consistent conditions, including treatment with a fresh batch of ATR inhibitor. The new data show minimal variability between biological replicates and are fully consistent with the other results in the study. These updated panels have now been incorporated into the main figures (**Figure 3I**).

Fig 3F - is the increase in EdU incorporation after complementation significant?

For figure 3I representative images should be added

The two previously published DLD1 CIP2A KO clones (Adam et al., 2021) displayed distinct MiDAS phenotypes while consistently showing complete loss of MUS81 foci upon CIP2A knockout (Figure EV3B, C, D). Because the clonal variability in MiDAS made the interpretation of these data unclear and did not allow us to draw a firm conclusion, we repeated the experiment using siRNA-mediated depletion of CIP2A. In this setting, we found that CIP2A plays a statistically significant but relatively minor role in MiDAS in DLD1 cells (**Figure 3C,D**). The complementation experiment was performed in one CIP2A KO clone (clone 7), which exhibited the strongest MiDAS defect. Given the clonal variability and the limited interpretability of this approach, we concluded that the complementation data do not substantially strengthen the

manuscript. The same applies to the metaphase spread analyses, which were performed only in CIP2A KO clone 7.

We have therefore removed both the complementation experiments, and the metaphase spread data from the manuscript. We have kept the analysis of the two KO clones with respect to MiDAS and MUS81 foci, which supports the conclusion that MUS81 recruitment is consistently dependent on CIP2A, whereas the impact on MiDAS is context dependent.

Reviewer #2 (Significance (Required)):

The data presented in the manuscript is of high quality but unfortunately does not present a big advance compared to current knowledge. Nevertheless, it is useful to have side-by-side comparison of different cell lines and conditions and IF localization studies. Given the therapeutic interest in the CIP2A-TopBP1 pathway it is important to get all the details right and researches with interest in DNA repair during mitosis will have interest in this work.

Moreover, in this manuscript the authors demonstrate that the impact of CIP2A disruption on MiDAS is variable across different cell lines-and even between individual clones. The concept of MiDAS is still clouded by considerable ambiguity, possibly due to earlier studies overstating the consequences of knockdown or knockout. It is therefore great that this manuscript presents clear, unbiased observations, highlighting both inter-cell line differences and the partial nature of the effects. This kind of nuanced reporting is valuable for the field.

Reviewer #3

Meroni and colleagues present evidence that CIP2A is required to recruit the SMX complex to sites of replication stress in mitotic cells. Whilst the data generated when using U2OS cells seems to support a role for CIP2A in recruiting the SMX complex to sites of replication stress to facilitate MiDAS, as the authors point out, this pathway is not conserved in DLD1 cells. Although the authors suggest that this discrepancy in the data may relate to the fact that U2OS cells are ALT positive and the DLD1 cells are not, there is no experimental evidence to support this hypothesis. It would have been nice if the authors had backed up this hypothesis with data relating to how CIP2A regulates the SMX-MiDAS pathway in other ALT positive and negative cell lines. Taken together, after reading this manuscript, I am left wondering whether CIP2A is really important for SMX-dependent MiDAS or whether it is phenomenon that is found in some commonly used cancer cell lines and not others. Whilst it is important to publish conflicting results as they can explain why some research labs can reproduce published data and others can't, I think this manuscript would benefit from assessment of the role of CIP2A in mediating the recruitment of the SMX complex to carry out MiDAS in a variety of additional cancer cell lines and also non-cancer cell lines, such as RPE1-hTERT cells to obtain some sort of consensus about the importance of CIP2A in dealing with mitotic replication stress.

We thank the reviewer for this thoughtful and constructive comment. We agree that the limited number of cell lines analyzed in the initial submission represented a weakness and that broader validation strengthens the conclusions. To address this point, we extended our analysis to four additional cell lines: non-transformed RPE1-hTERT and RPE1-hTERT p53 KO cells and the BRCA2-deficient pancreatic adenocarcinoma cell line CAPAN-1 and its BRCA2-complemented derivative. In CIP2A KO RPE1-hTERT cells and, we observed a mild but reproducible reduction in MiDAS after aphidicolin treatment. In contrast, no significant MiDAS defect was detected in the p53 KO CIP2A KO cells, consistent with previous findings by de Haan et al. 2025. Similarly, no MiDAS defect could be detected in CIP2A-depleted CAPAN-1 cells after aphidicolin treatment. These results indicate that the regulation of mitotic DNA synthesis is strongly influenced by cellular context and checkpoint status. Together with our data from U2OS and DLD1 cells, these results support the conclusion that the impact of CIP2A loss on MiDAS is context-dependent rather than universally conserved. Importantly, across all tested models, recruitment of MUS81 (and by extension the SMX complex) was consistently dependent on CIP2A following replication stress. We have incorporated these new data into the revised manuscript (**Figure 3E, F, G, H**) and adjusted the discussion to more clearly emphasize that SMX recruitment and MiDAS can be mechanistically separable, depending on cellular background.

Comments:

1. Fig.2A-E: Can the authors comment on the difference in number of APH-induced FANCD2, SLX4, Mus81 and XPF foci in mitotic U2OS cells? Given that SLX4 should be recruiting both XPF and Mus81, there is a disparity between the numbers of mitotic foci given that there are approximately 30 FANCD3 foci per mitotic cell following APH treatment. Additionally, why do the XPF foci not increase after APH exposure?

This is an interesting point. As the reviewer pointed out, our data shows significantly more FANCD2 foci than SLX4, MUS81, or XPF foci. This is consistent with the literature, as FANCD2 functions as a broad sensor and scaffolding protein involved in multiple types of DNA lesion repair. We therefore expect it to mark a wider range of lesions than the more specialized SMX complex, which is recruited for specific resolution pathways. As the reviewer correctly noted, SLX4 recruits MUS81 and XPF, so a similar number of foci is expected. This is indeed the case. However, numbers are slightly different, and this could be due to technical and biological reasons. Technically, the number of foci can be influenced by the specific antibody's affinity, avidity, and overall performance in immunofluorescence. This may lead to slight variations in detection efficiency between different protein targets. Biologically, the nucleases recruitment is a highly dynamic and fast process. Our data represents a static "snapshot" taken 15 minutes after mitotic release. The recruitment, assembly, and dismissal of these repair factors are transient and not necessarily perfectly synchronous, which can lead to small differences in the counted foci.

We have repeated the XPF experiment changing the IF condition, namely we included Triton-X during primary and secondary incubation, and this helped a lot in visualizing XPF foci. New data are now included as **Figure 2E**. As reported by others in the literature, we could clearly see an increase in XPF foci after aphidicolin treatment.

Moreover, to directly address whether CIP2A mediates recruitment of the SMX complex *per se*, we performed triple immunofluorescence staining for SLX4, XPF, and MUS81, taking advantage of antibodies raised in different species. We then quantified foci in which all three proteins colocalize using an automated CellProfiler pipeline, thereby restricting our analysis to sites likely representing assembled SMX complexes rather than individual subunits. Consistent with our single-subunit analyses, the number of triple-colocalizing foci was significantly reduced upon CIP2A depletion, following the same trend observed for the individual components (**Figure 2B, F**). These results support the conclusion that CIP2A promotes the recruitment of the SMX complex to mitotic DNA lesions.

2. Fig.2G: I would say that the 'full rescue' of Mus81 foci in the CIP2A KO cells complemented with WT CIP2A is not hugely convincing since there is only a difference of 1-2 foci between the WT and CIP2A KO cells treated with APH.

4. Fig.3H-I: Given that the reduction in MiDAS in the CIP2A KO cl.7 cell line is likely a clonal artifact not related to the loss of CIP2A, it is unclear how to interpret the data about the EdU foci pattern on chromosomes presented in Fig.3H-I and its relevance to CIP2A. Therefore, I am not sure this data really adds anything to the manuscript.

The two previously published DLD1 CIP2A KO clones (Adam et al., 2021) displayed distinct MiDAS phenotypes while consistently showing complete loss of MUS81 foci upon CIP2A knockout (**Figure EV3B, C, D**). Because the clonal variability in MiDAS made the interpretation of these data difficult and did not allow us to draw a firm conclusion, we repeated the experiment using siRNA-mediated depletion of CIP2A. In this setting, we found that CIP2A plays a statistically significant but relatively minor role in MiDAS in DLD1 cells while being crucial for MUS81 recruitment (**Figure 3C, D**). The complementation experiment was performed in one KO clone (clone 7), which exhibited the strongest MiDAS defect. Given the clonal variability and the limited interpretability of this approach, we concluded that the complementation data do not substantially strengthen the manuscript. The same applies to the metaphase spread analyses, which were performed only in CIP2A KO clone 7.

We have therefore removed both the complementation experiments, and the metaphase spread data from the manuscript. We have kept the analysis of the two KO clones with respect to MiDAS and MUS81 foci, which supports the conclusion that MUS81 recruitment is consistently dependent on CIP2A, whereas the impact on MiDAS is context dependent.

3. Fig.3A: I am not really sure how biologically meaningful a difference of 0.03-0.04 EdU foci per chromosome is when comparing BRCA2 KO DLD1 cells treated with control siRNA versus CIP2A siRNA. Would it not have been better to treat the BRCA2 KO DLD1 cells with APH?

We thank the reviewer for this comment. The difference may appear small because the values are reported as the number of EdU foci per chromosome, rather than per metaphase spread. We agree that this presentation may be misleading when compared with the immunofluorescence data, where foci are reported per mitotic cell. To facilitate comparison and avoid confusion, we have now replaced the graph with the one showing the number of EdU foci per metaphase spread (i.e., per cell). Because metaphase spreads can contain a variable number of chromosomes, and there is no unbiased way to ensure that all chromosomes from a given spread originate from the same cell or that none are lost during preparation, we also report the total number of chromosomes per spread as a control. This ensured that the results are not skewed by differences in

chromosome number. Our data are consistent with the immunofluorescence results recently reported by Martin et al. 2025. For this reason, we have moved these analyses to the Extended View figures (**Figure EV3F, G**).

5. Fig.4H: The difference in Mus81 foci per mitotic cell with/without the expression of B6L is only one focus per mitotic cell. Based on this, it is difficult to make any real conclusions about whether the TOPBP1-CIP2A interaction is really required for the recruitment of Mus81 to sites of mitotic replication stress.

We thank the reviewer for this comment and agree that the absolute difference in MUS81 foci per mitotic cell is numerically small. However, when examining the full data distribution rather than only the median values, a clear shift is evident: a substantial fraction of aphidicolin-treated control cells displays MUS81 foci (APH - red), whereas this fraction is markedly reduced upon disruption of CIP2A-TOPBP1 interaction (APH + S1 - purple) (see below - **Rev Figure 2A-B**). Thus, although the median difference corresponds to approximately one focus per mitotic cell, the effect reflects a consistent reduction in the proportion of cells showing MUS81 foci. We acknowledge that the overall number of aphidicolin-induced MUS81 foci in DLD1 B6L cells is low, which limits the magnitude of the measurable effect and makes interpretation based solely on absolute numbers challenging. Nevertheless, the observed reduction is reproducible across biological replicates and statistically significant. We agree that cell lines such as U2OS, which exhibit more robust MUS81 foci formation, might provide clearer results. However, the B6L system is currently established only in DLD1 cells. We have revised the text to temper our conclusions accordingly and to avoid overinterpretation of the magnitude of the effect.

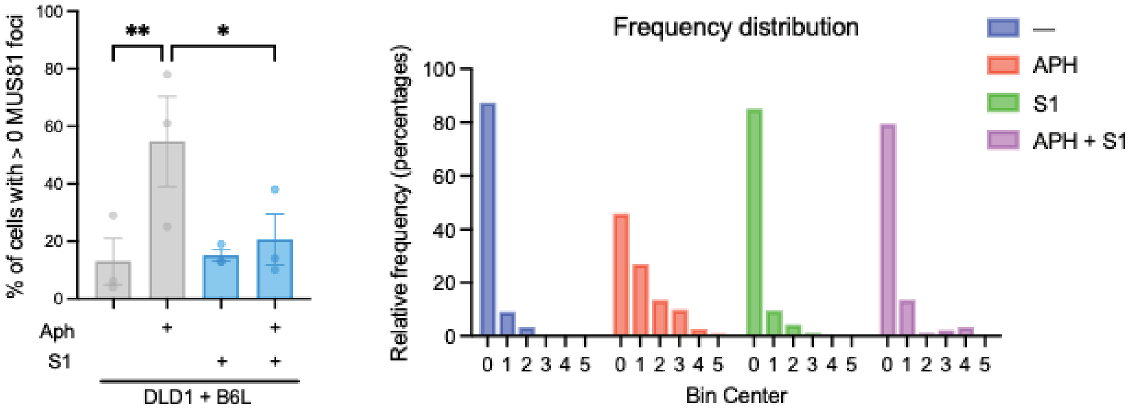

Rev Figure 2

Reviewer #3 (Significance (Required)):

As mentioned above, it is clear that the role of CIP2A in regulating the mitotic replication stress response by promoting recruitment of the SMX complex to sites of mitotic replication stress to promote MiDAS is complicated and may be specific to some cancer cell lines and not others. Whilst it is not clear what the underlying reason for this is, this manuscript would definitely benefit from additional analysis of this pathway in other cancer and non-cancer cell lines to obtain a consensus about the role of CIP2A.

This manuscript would appeal to fundamental research scientists interested in understanding the mechanisms underlying DNA damage repair, the replication stress response and mitotic regulation.

1st Revision - Editorial Decision 27th Apr 2026

Dear Manuel,

Thank you for the submission of your revised manuscript. We have now received the enclosed reports from the referees, and I am happy to say that all support its publication now. Only a few editorial requests will need to be addressed before we can proceed with the official acceptance of your manuscript.

- Please correct the conflict of interest subheading to "Disclosure and Competing Interests Statement"

- The author credits need to be removed from the ms file. All credits need to be entered during online ms submission.

- The reference format is not correct. It needs to be alphabetical and "et al" needs to be used after 10 author names. Please correct to the EMBO reports reference style.

- Please add a callout for Fig 4E to the ms text.

- Materials and methods needs to be corrected to 'Methods'

- The manuscript sections should be in the following order: Title page - Abstract - Introduction - Results - Discussion - Methods - Data Availability - Acknowledgments - Disclosure and Competing Interests Statement - References - Figure Legends - (Main Tables with legends if applicable) - Expanded View Figure Legends.

- Figure 2: panel H is missing in the caption

* Figure Legends - Comments *

- Please note that the figure 2H is mislabeled as figure 2E in the manuscript. This needs to be rectified.
- Please define the annotated p values ****/***/**/* as well as provide the exact p-values for the same in the legend of figure 1A, B, C, D, E, F, I, J; 2A, C, D, E, F, H; 3B-I; 4C-G; 5B-D; EV1 A, C, D, E; EV3 C, D, F, H; as appropriate and reasonable.
- Please note that the dotted borders are not defined in the legend of figure 1G, 2B, 3A. This needs to be rectified.

I would like to suggest some minor changes to the title and second half of the abstract. Please let me know whether your agree with this:

CIP2A recruits SLX4-MUS81-XPF in Mitosis and Protects Against Replication Stress

Here, we investigate the role of CIP2A in mitotic responses to replication stress. We show that replication stress induces a strong increase in CIP2A foci during mitosis, highlighting its involvement in processing under-replicated DNA. In wild-type cells, CIP2A is required for efficient recruitment of the scaffold SLX4 and the nucleases MUS81 and XPF to sites of under-replicated DNA (OK?). CIP2A loss disrupts this recruitment and leads to increased anaphase lagging chromosomes and micronuclei formation. In a subset of cell lines, CIP2A is also required for mitotic DNA synthesis (MiDAS), although MiDAS and nuclease recruitment can be genetically separable (the meaning here is unclear, at least to me. Better re-write).
Together, our findings identify CIP2A as a regulator of mitotic processing of under-replicated DNA and provide a framework for understanding context-dependent vulnerabilities in cancer cells.

EMBO press papers are accompanied online by A) a short (1-2 sentences) summary of the findings and their significance, B) 2-3 bullet points highlighting key results and C) a synopsis image that is exactly 550 pixels wide and 300 pixels high. The synopsis image should provide a sketch of the major findings, like a graphical abstract. Please note that text needs to be readable at the final size. Please send us this information along with the final manuscript.

I look forward to seeing a final version of your manuscript as soon as possible.

Best regards,
Esther

Esther Schnapp, PhD
Senior Editor
EMBO reports

Referee #1:

The authors have adequately addressed my concerns and the revised manuscript is substantially improved. I welcome the presentation of focus quantification with superplots but would have preferred to have single cells color-coded according to biological replicate.

Referee #2:

The authors have adequately addressed my comments. I would now recommend the manuscript for publication in EMBO Reports.

Referee #3:

The authors of addressed the majority of my comments satisfactorily. I recommend for them to do another review of the citations prior to publication, particularly in the introduction as there are still some inaccuracies (this is the minor revision).

All minor editorial requests have been addressed by the authors.

Prof. Manuel Stucki
University of Zurich
Dptm of Gynaecology
Wagistrasse 14
Schlieren 8952
Switzerland

Dear Manuel,

I am very pleased to accept your manuscript for publication in the next available issue of EMBO reports. Thank you for your contribution to our journal.

Your manuscript will be processed for publication by EMBO Press. It will be copy edited and you will receive page proofs prior to publication. Please note that you will be contacted by Springer Nature Author Services to complete licensing and payment information.

You may qualify for financial assistance for your publication charges - either via a Springer Nature fully open access agreement or an EMBO initiative. Check your eligibility: https://link.springer.com/journal/44319/how-to-publish-with-us

Should you be planning a Press Release on your article, please get in contact with embo_production@springernature.com as early as possible in order to coordinate publication and release dates.

If you have any questions, please do not hesitate to contact the Editorial Office. Thank you for your contribution to EMBO Reports.

Best regards,
Esther

Esther Schnapp, PhD
Senior Editor
EMBO reports

>>> Please note that it is EMBO Reports policy for the transcript of the editorial process (containing referee reports and your response letter) to be published as an online supplement to each paper. If you do NOT want this, you will need to inform the Editorial Office via email immediately. More information is available here: https://link.springer.com/partners/embo-press/editorial-policies#Peer%20review

EMBO Press Author Checklist

Corresponding Author Name: Manuel Stucki, Alice Meroni	
Journal Submitted to: EMBO Reports	
Manuscript Number: EMBOR-2025-63387-T	

USEFUL LINKS FOR COMPLETING THIS FORM
The EMBO Journal - Author Guidelines
EMBO Reports - Author Guidelines
Molecular Systems Biology - Author Guidelines
EMBO Molecular Medicine - Author Guidelines

Reporting Checklist for Life Science Articles (updated January
This checklist is adapted from Materials Design Analysis Reporting (MDAR) Checklist for Authors. MDAR establishes a minimum set of requirements in transparent reporting in the life sciences (see Statement of Task: 10.31222/osf.io/9sm4x). Please follow the journal's guidelines in preparing your
Please note that a copy of this checklist will be published alongside your article.

Abridged guidelines for figures
1. Data
The data shown in figures should satisfy the following conditions:
- the data were obtained and processed according to the field's best practice and are presented to reflect the results of the experiments in an accurate and unbiased manner.
- ideally, figure panels should include only measurements that are directly comparable to each other and obtained with the same assay.
- plots include clearly labeled error bars for independent experiments and sample sizes. Unless justified, error bars should not be shown for technical
- if n<5, the individual data points from each experiment should be plotted. Any statistical test employed should be justified.
- Source Data should be included to report the data underlying figures according to the guidelines set out in the authorship guidelines on Data

2. Captions
Each figure caption should contain the following information, for each panel where they are relevant:
- a specification of the experimental system investigated (eg cell line, species name).
- the assay(s) and method(s) used to carry out the reported observations and measurements.
- an explicit mention of the biological and chemical entity(ies) that are being measured.
- an explicit mention of the biological and chemical entity(ies) that are altered/varied/perturbed in a controlled manner.
- the exact sample size (n) for each experimental group/condition, given as a number, not a range;
- a description of the sample collection allowing the reader to understand whether the samples represent technical or biological replicates (including how many animals, litters, cultures, etc.).
- a statement of how many times the experiment shown was independently replicated in the laboratory.
- definitions of statistical methods and measures:
  - common tests, such as t-test (please specify whether paired vs. unpaired), simple χ2 tests, Wilcoxon and Mann-Whitney tests, can be unambiguously identified by name only, but more complex techniques should be described in the methods section;
  - are tests one-sided or two-sided?
  - are there adjustments for multiple comparisons?
  - exact statistical test results, e.g., P values = x but not P values < x;
  - definition of 'center values' as median or average;
  - definition of error bars as s.d. or s.e.m.

Please complete ALL of the questions below.
Select "Not Applicable" only when the requested information is not relevant for your study.

Materials

Newly Created Materials	Information included in the manuscript?	In which section is the information available? (Reagents and Tools Table, Materials and Methods, Figures, Data Availability Section)
New materials and reagents need to be available; do any restrictions apply?	Not Applicable	

Antibodies	Information included in the manuscript?	In which section is the information available? (Reagents and Tools Table, Materials and Methods, Figures, Data Availability Section)
For **antibodies** provide the following information:<br>- Commercial antibodies: RRID (if possible) or supplier name, catalogue number and or/clone number<br>- Non-commercial: RRID or citation	Yes	Reagents and Tools Table<br>Materials and Methods

DNA and RNA sequences	Information included in the manuscript?	In which section is the information available? (Reagents and Tools Table, Materials and Methods, Figures, Data Availability Section)
**Short novel DNA or RNA including primers, probes**: provide the sequences.	Yes	Reagents and Tools Table

Cell materials	Information included in the manuscript?	In which section is the information available? (Reagents and Tools Table, Materials and Methods, Figures, Data Availability Section)
**Cell lines:** Provide species information, strain. Provide accession number in repository **OR** supplier name, catalog number, clone number, and/**OR** RRID.	Yes	Reagents and Tools Table
**Primary cultures:** Provide species, strain, sex of origin, genetic modification status.	Not Applicable	
Report if the cell lines were recently **authenticated** (e.g., by STR profiling) and tested for mycoplasma contamination.	Yes	Materials and Methods

Experimental animals	Information included in the manuscript?	In which section is the information available? (Reagents and Tools Table, Materials and Methods, Figures, Data Availability Section)
**Laboratory animals or Model organisms:** Provide species, strain, sex, age, genetic modification status. Provide accession number in repository **OR** supplier name, catalog number, clone number, **OR** RRID.	Not Applicable	
**Animal observed in or captured from the field:** Provide species, sex, and age where possible.	Not Applicable	
Please detail **housing and husbandry conditions**.	Not Applicable	

Plants and microbes	Information included in the manuscript?	In which section is the information available? (Reagents and Tools Table, Materials and Methods, Figures, Data Availability Section)
**Plants:** provide species and strain, ecotype and cultivar where relevant, unique accession number if available, and source (including location for collected wild specimens).	Not Applicable	
**Microbes:** provide species and strain, unique accession number if available, and source.	Not Applicable	

Human research participants	Information included in the manuscript?	In which section is the information available? (Reagents and Tools Table, Materials and Methods, Figures, Data Availability Section)
If collected and within the bounds of privacy constraints report on age, sex and gender or ethnicity for all study participants.	Not Applicable	

Core facilities	Information included in the manuscript?	In which section is the information available? (Reagents and Tools Table, Materials and Methods, Figures, Data Availability Section)
If your work benefited from core facilities, was their service mentioned in the acknowledgments section?	Yes	Materials and Methods

Design

Study protocol	Information included in the manuscript?	In which section is the information available? (Reagents and Tools Table, Materials and Methods, Figures, Data Availability Section)
If study protocol has been **pre-registered, provide DOI in the manuscript**. For clinical trials, provide the trial registration number **OR** cite DOI.	Not Applicable	
Report the **clinical trial registration number** (at ClinicalTrials.gov or equivalent), where applicable.	Not Applicable	

Laboratory protocol	Information included in the manuscript?	In which section is the information available? (Reagents and Tools Table, Materials and Methods, Figures, Data Availability Section)
Provide DOI OR other citation details if **external detailed step-by-step protocols** are available.	Not Applicable	

Experimental study design and statistics	Information included in the manuscript?	In which section is the information available? (Reagents and Tools Table, Materials and Methods, Figures, Data Availability Section)
Include a statement about **sample size** estimate even if no statistical methods were used.	Yes	Materials and Methods
Were any steps taken to minimize the effects of subjective bias when allocating animals/samples to treatment (e.g. **randomization procedure**)? If yes, have they been described?	Not Applicable	
Include a statement about **blinding** even if no blinding was done.	Yes	Materials and Methods
Describe **inclusion/exclusion criteria** if samples or animals were excluded from the analysis. Were the criteria pre-established?<br><br>If sample or data points were omitted from analysis, report if this was due to attrition or intentional exclusion and provide justification.	Not Applicable	
For every figure, are **statistical tests** justified as appropriate? Do the data meet the assumptions of the tests (e.g., normal distribution)? Describe any methods used to assess it. Is there an estimate of variation within each group of data? Is the variance similar between the groups that are being statistically compared?	Yes	Materials and Methods

Sample definition and in-laboratory replication	Information included in the manuscript?	In which section is the information available? (Reagents and Tools Table, Materials and Methods, Figures, Data Availability Section)
In the figure legends: state number of times the experiment was **replicated** in laboratory.	Yes	Materials and Methods and Figures
In the figure legends: define whether data describe **technical or biological replicates**.	Yes	Materials and Methods and Figures

Ethics

Ethics	Information included in the manuscript?	In which section is the information available? (Reagents and Tools Table, Materials and Methods, Figures, Data Availability Section)
Studies involving **human participants**: State details of **authority granting ethics approval** (IRB or equivalent committee(s), provide reference number for approval.	Not Applicable	
Studies involving **human participants**: Include a statement confirming that **informed consent** was obtained from all subjects and that the experiments conformed to the principles set out in the WMA Declaration of Helsinki and the Department of Health and Human Services Belmont Report.	Not Applicable	
Studies involving **human participants:** For publication of **patient photos**, include a statement confirming that consent to publish was obtained.	Not Applicable	
Studies involving experimental **animals**: State details of **authority granting ethics approva**l (IRB or equivalent committee(s), provide reference number for approval. Include a statement of compliance with ethical regulations.	Not Applicable	
Studies involving **specimen and field samples:** State if relevant **permits** obtained, provide details of authority approving study; if none were required, explain why.	Not Applicable	

Dual Use Research of Concern (DURC)	Information included in the manuscript?	In which section is the information available? (Reagents and Tools Table, Materials and Methods, Figures, Data Availability Section)
Could your study fall under dual use research restrictions? Please check biosecurity documents and list of **select agents and toxins** (CDC): https://www.selectagents.gov/sat/list.htm	Not Applicable	
If you used a select agent, is the security level of the lab appropriate and reported in the manuscript?	Not Applicable	
If a study is subject to dual use research of concern regulations, is the name of the **authority granting approval and reference number** for the regulatory approval provided in the manuscript?	Not Applicable	

Reporting

The MDAR framework recommends adoption of discipline-specific guidelines, established and endorsed through community initiatives. Journals have their own policy about requiring specific guidelines and recommendations to complement MDAR.

Adherence to community standards	Information included in the manuscript?	In which section is the information available? (Reagents and Tools Table, Materials and Methods, Figures, Data Availability Section)
State if relevant guidelines or checklists (e.g., **ICMJE, MIBBI, ARRIVE, PRISMA**) have been followed or provided.	Not Applicable	
For **tumor marker prognostic studies,** we recommend that you follow the **REMARK** reporting guidelines (see link list at top right). See author guidelines, under 'Reporting Guidelines'. Please confirm you have followed these guidelines.	Not Applicable	
For **phase II and III randomized controlled trials**, please refer to the **CONSORT** flow diagram (see link list at top right) and submit the CONSORT checklist (see link list at top right) with your submission. See author guidelines, under 'Reporting Guidelines'. Please confirm you have submitted this list.	Not Applicable	

Data Availability

Data availability	Information included in the manuscript?	In which section is the information available? (Reagents and Tools Table, Materials and Methods, Figures, Data Availability Section)
Have **primary datasets** been deposited according to the journal's guidelines (see 'Data Deposition' section) and the respective accession numbers provided in the Data Availability Section?	Not Applicable	
Were **human clinical and genomic datasets** deposited in a public access-controlled repository in accordance to ethical obligations to the patients and to the applicable consent agreement?	Not Applicable	
Are **computational models** that are central and integral to a study available without restrictions in a machine-readable form? Were the relevant accession numbers or links provided?	Not Applicable	
If publicly available data were reused, provide the respective **data citations in the reference list**.	Not Applicable	
